# Blood Pressure and Late Pregnancy Circulating miRNAs in the MADRES Study

**DOI:** 10.1161/JAHA.124.040416

**Published:** 2025-06-11

**Authors:** Elizabeth C. Anderson, Meghan E. Muse, Zhongzheng Niu, Helen B. Foley, Joshua J. Levy, Megan E. Romano, Jiang Gui, Jessica L. Bentz, Shohreh F. Farzan, Theresa M. Bastain, Carmen J. Marsit, Carrie V. Breton, Caitlin G. Howe

**Affiliations:** ^1^ Department of Epidemiology Geisel School of Medicine at Dartmouth Lebanon NH; ^2^ Division of Environmental Health, Department of Population and Public Health Sciences, Keck School of Medicine University of Southern California Los Angeles CA; ^3^ Emerging Diagnostic and Investigative Technologies, Department of Pathology and Laboratory Medicine Dartmouth Hitchcock Medical Center Lebanon NH; ^4^ Department of Pathology and Laboratory Medicine and the Department of Computational Biomedicine Cedars‐Sinai Medical Center Los Angeles CA; ^5^ Department of Biomedical Data Science Geisel School of Medicine at Dartmouth Hanover NH; ^6^ Department of Pathology Dartmouth‐Hitchcock Medical Center Lebanon NH; ^7^ Gangarosa Department of Environmental Health, Rollins School of Public Health Emory University Atlanta GA

**Keywords:** blood pressure, EVP miRNAs, hypertension, placenta, pregnancy, Pregnancy, Epidemiology, Epigenetics, Preeclampsia, High Blood Pressure

## Abstract

**Background:**

Circulating extracellular and vesicle particle (EVP) miRNAs have been associated with cardiovascular risk and adverse birth outcomes. Hypertensive disorders of pregnancy (HDP) increase risk for adverse birth outcomes and future cardiovascular outcomes in mothers and children and have been associated with altered maternal circulating EVP miRNA levels during pregnancy. Whether these relationships exist for elevated blood pressure (BP) in the subclinical range is unknown. We investigated associations between (1) hypertensive disorders of pregnancy and (2) maternal BP trajectories, including in the subclinical range, and circulating EVP miRNA levels during pregnancy in the MADRES (Maternal and Developmental Risks From Environmental and Social Stressors) Study (n=372).

**Methods:**

Latent class trajectory modeling was used to identify trajectories from BP measures abstracted from medical records. The NanoString nCounter platform was used to quantify 798 miRNAs extracted from maternal blood (median gestational age: 31.6 weeks). Covariate‐adjusted regression models assessed associations between each hypertensive disorders of pregnancy subtype or BP trajectory and levels of each miRNA.

**Results:**

Three BP trajectories were identified: Low, Moderate, and High. Chronic hypertension was associated with higher levels of miR‐1185‐2‐3p (*P*_false discovery rate_<0.05), a placenta‐specific miRNA linked to arterial stiffness and preterm delivery. Many placenta‐expressed miRNAs previously associated with a longer gestational duration in the same cohort were lower among participants with elevated BP (*P*<0.05). Target genes of BP‐associated EVP miRNAs were overrepresented in pathways involved in vascular inflammation, oxidative stress, endothelial dysfunction, and placental function.

**Conclusions:**

Circulating levels of placenta‐expressed EVP miRNAs previously implicated in adverse birth and cardiovascular outcomes are sensitive to elevated maternal BP during pregnancy, including in the subclinical range.

## Graphical abstract

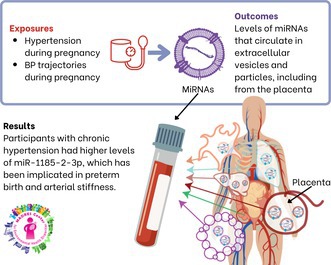

**Nonstandard Abbreviations and Acronyms**
C19MC: chromosome 19 microRNA cluster
C14MC: chromosome 14 microRNA cluster
EVP: extracellular vesicles and particles
HDP: hypertensive disorders of pregnancy
MADRES: Maternal and Developmental Risks From Environmental and Social Stressors Study

**Research Perspective**

**What Is New?**

- In a prospective pregnancy cohort with a large panel of extracellular vesicle and particle miRNAs profiled in maternal plasma in late pregnancy, we found that participants with chronic hypertension had elevated circulating levels of an miRNA (miR‐1185‐2‐3p) that has been implicated in preterm delivery and arterial stiffness.
- Using latent class trajectory modeling, we identified a subgroup of participants with persistently elevated subclinical blood pressure across pregnancy who had lower circulating levels of multiple extracellular vesicle and particle miRNAs important for placental function that were previously associated with a longer gestational duration in the same cohort.

**What Question Should Be Addressed Next?**

- Future research is needed to determine whether circulating extracellular vesicle and particle miRNAs associated with elevated blood pressure during pregnancy can serve as biomarkers of postpartum cardiovascular health and other adverse maternal and child health outcomes.

Hypertensive disorders of pregnancy (HDP) increase the risk for complications during pregnancy and delivery^1^, ^2^, ^3^, ^4^, ^5^ and long‐term cardiovascular outcomes in both mothers^6^, ^7^, ^8^ and children.^9^, ^10^, ^11^ Although HDP reflect a group of heterogeneous diseases that differ in their time of onset or organ involvement, they are all characterized by elevated blood pressure (BP), defined by the American College of Obstetrics as systolic BP (SBP) ≥140 mm Hg or diastolic BP ≥90.^12^, ^13^ Growing evidence suggests that even in the subclinical range (ie, below these cutoffs), elevated BP may increase risk for the same complications associated with overt hypertension.^14^, ^15^, ^16^, ^17^

Modeling longitudinal BP measures during gestation using trajectory approaches, such as latent class trajectory modeling (LCTM), may offer more nuanced insight into the potential influences of elevated BP on both maternal and child health than using information on HDP diagnosis alone. This approach may be especially useful for capturing information on elevated BP within the subclinical range and changes in BP across pregnancy, which may be clinically meaningful. Prior research has suggested that a typical BP trajectory during pregnancy is characterized by BP measures that gradually decrease until ~20 weeks gestation, then moderately increase until delivery.^18^ However, not everyone follows this pattern, and even within this broad trajectory there may be subgroups that start and end their pregnancies with much higher or lower levels of BP. A major advantage of modeling BP trajectories across gestation is the ability to identify these distinct subgroups, which have increasingly been associated with adverse birth outcomes and subsequent cardiovascular risk.^17^, ^19^, ^20^, ^21^ However, little is known about the molecular mechanisms underlying these effects.

Circulating miRNAs hold promise as minimally invasive biomarkers of adverse cardiovascular outcomes.^22^, ^23^, ^24^ For example, in nonpregnant individuals, circulating levels of miRNAs have been identified as potential prognostic biomarkers or therapeutic targets for hypertension,^25^, ^26^, ^27^ or have been implicated in processes that contribute to atherosclerosis, such as endothelial cell apoptosis and inflammation.^22^, ^23^, ^28^ However, whether these (or other) miRNAs may be similarly useful indicators of future cardiovascular risk in pregnant populations is unknown, as circulating miRNA profiles change during gestation. This is partly due to miRNA release from the placenta,^24^ which facilitate maternal‐offspring communication.^29^, ^30^, ^31^ These include miRNAs from the chromosome 14 and 19 clusters (C14MC/C19MC), which are almost exclusively expressed in the placenta and may provide unique insights into placental health and function during pregnancy.^32^, ^33^

Growing evidence supports that maternal circulating miRNAs respond to adverse in utero environments. Several case–control studies have reported associations between HDP and levels of maternal circulating miRNAs during pregnancy,^34^, ^35^, ^36^, ^37^, ^38^, ^39^, ^40^, ^41^, ^42^, ^43^ many of which are involved in apoptosis, angiogenesis, and inflammation, which are pathways important for placental development and future cardiovascular risk. These findings suggest that elevated BP may be associated with maternal circulating miRNAs during pregnancy, a critical period during which risk for future cardiovascular diseases can be unmasked.^44^ A better understanding of the relationships between elevated BP and circulating miRNAs may therefore provide insight into susceptibility to adverse birth outcomes and postpartum cardiovascular disease.^45^, ^46^, ^47^ Notably, prior research has predominantly focused on preeclampsia and specific candidate miRNAs of interest with small sample sizes. Thus, larger studies that assess the impacts of different HDP subtypes, as well as elevated BP in the subclinical range, on circulating miRNAs during pregnancy are needed.

We addressed this gap using data from the MADRES (Maternal and Developmental Risks From Environmental and Social Stressors) study. In addition to information on HDP, rich longitudinal BP data are available during pregnancy. Three unique BP trajectories were previously identified from these data using LCTM, including a subgroup reflecting subclinical elevated BP across pregnancy, which was associated with a higher risk for postpartum hypertension.^17^ Given these findings, and findings from prior studies focused on overt hypertension, we hypothesized that higher maternal BP in both the clinical and subclinical range alters circulating miRNAs that are known to be involved in vascular endothelial function, endothelial cell apoptosis, inflammation, hypoxia, angiogenesis, and placental development and function.

## METHODS

### Study Participants

MADRES is a longitudinal pregnancy cohort^48^ that launched in 2015 and enrolled 1065 mother–child pairs. Participants were recruited from prenatal providers that primarily care for medically underserved populations in Los Angeles, California, and are predominantly low‐income and Hispanic. Eligibility criteria included being <30 weeks gestation and ≥18 years of age at the time of enrollment, speaking English or Spanish fluently, not currently incarcerated, having a singleton pregnancy, an HIV‐negative status, and the ability to provide informed consent. The current study focused on a subset of MADRES participants (n=372) with at least 1P measure in their electronic medical records (EMRs) during pregnancy and late pregnancy measures of maternal circulating miRNAs available (Figure S1). Selection of participants for miRNA profiling was guided by aims from prior research^49^, ^50^; the subset of selected participants were similar to those not selected on most characteristics (Table1).

**Table 1:** Participants Excluded Versus Included From the Analysis

Characteristic	Participants excluded from analyses, n=693	Participants included in analyses, n=372	*P* value*
Maternal age, y	27.4 (18–45.1)	28.6 (18.3–45.5)	0.292
Prepregnancy body mass index, kg/m^2^	27.8 (15.6–55.6)	27.8 (17.6–60.6)	0.755
Center for Epidemiologic Studies–Depression Scale	8 (0–35)	8 (0–46)	0.821
Perceived Stress Scale	13 (0–25)	12 (0–32)	0.025‡
Pregnancy Physical Activity Questionnaire, metabolic equivalent task‐hours per week	231 (25.3–839.6)	250.8 (22.8–813)	0.056
Number of systolic blood pressure measures during pregnancy	11 (1–30)	14 (1–34)	1.588×10^−19^ ‡
Gestational age at delivery, wks	39.3 (23.4–42.1)	39.3 (33.6–42.4)	0.758
Gestational weight gain, kg	11.5 (−29.5–48.5)	10.7 (−30.4–43.6)	0.294
Birth order of child	2 (1–6)	2 (1–6)	0.224
Enrolled at ≥20 wks gestation	179 (25.8%)	95 (25.5%)	0.976
Any diagnosed diabetes			
Yes	173 (25.0%)	131 (35.2%)	0.580
No	348 (50.2%)	241 (64.8%)	
Missing	172 (24.8%)	0 (0%)	
Reported prenatal vitamin use†			
Yes	473 (68.3%)	363 (97.6%)	0.220
No	19 (2.7%)	8 (2.2%)	
Missing	201 (29.0%)	1 (0.3%)	
Infant sex			
Female	281 (40.5%)	178 (47.8%)	0.197
Male	255 (36.8%)	194 (52.2%)	
Missing	157 (22.7%)	0 (0.0%)	
HDP subtype			
No HDP	411 (59.3%)	300 (80.6%)	0.568
Preeclampsia/eclampsia	65 (9.4%)	41 (11.0%)	
Chronic hypertension	16 (2.3%)	10 (2.7%)	
Gestational hypertension	41 (5.9%)	21 (5.6%)	
Missing	160 (23.1%)	0 (0.0%)	
Maternal education			
≤12th grade	354 (51.1%)	213 (57.2%)	0.187
>12th grade	258 (37.2%)	155 (41.7%)	
Missing	81 (11.7%)	4 (1.1%)	
Smoking			
Did not smoke during pregnancy	480 (69.3%)	365 (98.1%)	0.615
Smoked during pregnancy	13 (1.9%)	7 (1.9%)	
Missing	200 (28.9%)	0 (0%)	

Values are median (range) or N (%). HDP indicates hypertensive disorders of pregnancy.

* Statistical comparisons for continuous variables were completed with the Wilcoxon rank sum test and for categorical variables with Pearson's chi‐square test. “Missing” categories were excluded from statistical comparisons to ensure a fair comparison.

^†^ Missing and “no” responses were combined due to only 1 participant having missing information in the analytic subset.

^‡^ *P*<0.05.

Written informed consent and HIPAA authorization to obtain medical records from each participant were obtained at study entry for each participant. The protocol was approved by the University of Southern California's Institutional Review Board. The data that support the findings of this study are available upon reasonable request.

### BP Trajectories and HDP Classification

In the analytic subset, 19.4% of participants had any HDP (ie, preeclampsia/eclampsia, chronic hypertension, or gestational hypertension), exceeding the national US prevalence of 13% to 16% during a similar time frame (2017–2019).^51^ Preeclampsia/eclampsia was classified based on physician diagnoses in EMRs. Among participants classified as having chronic hypertension or gestational hypertension, 61% (n=11) and 62% (n=13), respectively, were classified based on a physician diagnosis in the EMRs. Remaining participants were classified based on BP measures from EMRs using the American College of Obstetricians and Gynecologist guidelines.^13^

In addition to examining the relationships between HDP and circulating extracellular vesicle and particle (EVP) miRNAs, we also evaluated how BP trajectories across pregnancy relate to these miRNAs, with a particular interest in trajectories reflecting elevated BP within the subclinical range. To achieve this, BP trajectories were identified using continuous longitudinal BP measures from EMRs for all MADRES participants with data as of July 16, 2023. A median of 13 (interquartile range, 9–16) SBP measures per participant were available during pregnancy (Table1). We focused on SBP measures because (1) SBP is more sensitive to changes in the environment compared with diastolic BP and may be a better predictor of future hypertension risk^52^, ^53^, ^54^ and (2) diastolic BP trajectories showed limited variability during pregnancy (Figure S2). Before conducting LCTM, medical records with missing gestational age at BP measurement were excluded, as were outliers for either SBP or gestational age at SBP assessment (SBP>240 or ≤60 mm Hg; gestational age <21 days), representing 58 (0.54%) BP measures. A total of n=10 743 BP measures from n=854 MADRES participants who had at least 1 SBP measure (98% had a minimum of 3 measures available during pregnancy) were used.

BP trajectories were identified using LCTM, which is an unsupervised analytic approach. LCTM was conducted using the systematic framework proposed by Lennon et al.^55^ The LCMM (v 2.0.2) R package^56^ was used to compute latent BP classes. Previously, in a smaller sample of MADRES participants (n=732), our group used this same approach and identified 3 trajectories to be optimal.^17^ At the time of this analysis, a larger number of MADRES participants had longitudinal BP data available during pregnancy (n=854). We therefore recreated BP trajectories for this larger sample size. Consistent with our prior study, to determine the optimal number of trajectories (k), models with 1 to 5 trajectories were tested, and as recommended by Lennon et al, the model with the lowest Bayesian inference criterion was selected.^55^ The lowest Bayesian inference criterion was observed for k=3 trajectories (Table S1). Next, as recommended,^55^ we selected the favored k=3 model from 4 model refinements with various random slope and variance structures. As per the framework proposed by Lennon et al,^52^ the favored model was primarily selected based on the Bayesian inference criterion, which was the lowest for the model that included linear and quadratic random slopes for gestational age and a class‐specific variance–covariance structure. The odds of correct classification (ratio>5), mismatch between class proportions and membership (values close to 0 preferred), the distribution of participants across latent classes (>5%), and the average of maximum probabilities (>0.7) were also evaluated, as recommended. All of these model adequacy assessments were comparable to or superior for the favored model than other models (Table S1). Because LCTM results can be sensitive to these modeling decisions, we conducted sensitivity analyses comparing results for the primary selected model, which for the selection of “k” included a random linear and quadratic slope for the gestational age of BP measurement, with a model that excluded these random slopes to identify trajectories reflecting less interindividual heterogeneity.

### Maternal Blood Collection and miRNA Extraction and Quantification

Methods for extracting and profiling maternal circulating miRNAs in MADRES have been described previously.^50^, ^57^, ^58^ Briefly, during the third trimester (median=31.6 [interquartile range, 30.3–33.0] weeks) participants were asked to fast for 24 hours before collection of peripheral blood samples by a trained phlebotomist. Within 2 hours, these samples were transported on ice to the Southern California Environmental Health Sciences Center laboratory, fractionated, and stored at −80 °C. MiRNAs were extracted from 500 μL of plasma using the Qiagen ExoRNeasy kit and quantified by BioAnalyzer using a small RNA kit (Agilent Technologies, Inc. USA). Althoughthis kit is designed to enrich for extracellular vesicles, it may also capture other small miRNA‐carrying particles such as lipoproteins, as described previously.^59^ Therefore, we refer to the isolated material as EVPs hereafter. Samples with a minimum concentration of 100 pg/μL and a visible peak between 10 and 24 nucleotides were used for miRNA profiling. A total of 798 miRNAs were quantified by the University of Southern California Genomics Core using the NanoString nCounter Human v3 miRNA expression assay (Nanostring Technologies, Inc.), which measures >95% of all human miRbase reads and 100% of high‐confidence human miRNAs.^60^

As described previously,^50^, ^58^ miRNA counts were normalized to sample‐specific positive controls included on the nCounter platform using the NanoStringNorm R package.^61^ After normalization, 90 miRNAs were widely detectable (ie, counts>mean+1.5 SD of negative controls for >60% of participants), which were the primary outcomes. During normalization, 6 participants' samples were excluded due to unusually low miRNA counts (NanoStringNorm normalization factor>6). An additional 6 participants were extreme outliers based on total normalized miRNA counts (>mean+3×SD) and therefore excluded along with 5 participants recruited into MADRES through self‐referral from advertisements rather than from a prenatal clinic. After these steps, 372 participants remained in our analytic sample, all of whom had paired BP measures during pregnancy and relevant covariate data (Figure S1). None of the included samples exhibited evidence of hemolysis based on a color reference or levels of miRNAs identified as reliable biomarkers of hemolysis during pregnancy.^62^ Following these preprocessing steps, we corrected for batch effects of NanoString chip using ComBat (R package “sva”, v3.46.0).^63^

Many EVPs in circulation during pregnancy originate in placental tissue,^24^, ^32^, ^33^ and the C14MC/C19MC miRNAs are almost exclusively expressed in the placenta.^64^, ^65^ These miRNAs play important roles in pregnancy, have previously been associated with the pathogenesis of preeclampsia,^66^, ^67^ and have been identified in maternal circulation during pregnancy.^33^, ^66^, ^68^, ^69^, ^70^, ^71^, ^72^ We identified 115 miRNAs on the NanoString nCounter platform from the C14MC/C19MC, 7 of which met detection thresholds to be assessed as primary outcomes. Given prior evidence that hypertension during pregnancy alters placenta‐derived miRNAs,^73^, ^74^ we evaluated an additional 26 miRNAs from C14MC/C19MC that did not meet these detection thresholds but that were above detection for ≥20% of participants, modeled as binary variables (ie, detect versus nondetect) as secondary outcomes.

### Covariates

Covariates were abstracted from maternal EMRs or collected through questionnaires administered in Spanish or English. Key covariates were identified using directed acyclic graphs based on a priori knowledge (Figure S3). The minimal adjustment set identified from the directed acyclic graphs included maternal psychological stress during pregnancy, age, prepregnancy body mass index (BMI), smoking during pregnancy, and prenatal vitamin use. Maternal psychological stress during pregnancy was measured using the Perceived Stress Scale questionnaire. Information on maternal age, vitamin use, and smoking were collected by questionnaires administered at enrollment, and maternal prepregnancy BMI (kg/m^2^) was calculated using the participant's measured height and self‐reported prepregnancy weight. Only 7 participants reported smoking during pregnancy, so it was not included in primary models but was evaluated in sensitivity analyses. In addition to the minimal adjustment set of potential confounders identified by the directed acyclic graphs, 2 variables related to study design were included in all models: recruitment site and timing of enrollment (<20 or ≥20 weeks of gestation). The final covariates for all models were maternal age, perceived stress during pregnancy, BMI, recruitment site, and timing of enrollment, none of which had missing data in the analytic subset.

Given that the major determinants of maternal circulating EVP miRNAs during pregnancy are still largely unknown, additional potential confounders and precision variables were also identified using a principal component analysis approach, as described previously.^50^, ^57^, ^58^ Briefly, principal component analysis on the EVP miRNA data, was conducted using the R function prcomp included in the Stats package (v 4.2.2)^75^ (Figure S4). Associations between all covariates included in the directed acyclic graphs and study design variables were evaluated in relation to this first principal component (Table S2). One variable (maternal physical activity during pregnancy) that was not included in the minimal adjustment set was found to be associated with the first principal component and therefore considered in sensitivity analyses. To capture information about physical activity during pregnancy, we used responses from the validated Pregnancy Physical Activity Questionnaire^76^ administered during the participant's first study visit.

In sensitivity analyses, we evaluated the impact of additionally adjusting for (1) gestational age at blood draw and (2) total gestational weight, as greater weight gain. Total weight gain during pregnancy was calculated by subtracting the last recorded weight (up to 2 weeks after delivery) and the prepregnancy weight, collected by questionnaires or abstracted from EMRs. We also evaluated whether effect estimates for miRNAs associated (*P*<0.05) with an HDP subtype or BP trajectory were similar after excluding participants with (1) chronic hypertension (n=10), (2) gestational diabetes or type 2 diabetes (n=51), (3) a nonfasting status before blood collection (n=6), (4), no report of using prenatal vitamins (n=9), or (5) BP medication use (n=31). Diabetes status during pregnancy was abstracted from the maternal EMRs and questionnaires, as described previously.^77^ Information on prenatal vitamin use and BP medication use during pregnancy were collected by questionnaire.

### Statistical Analysis

Statistical analyses were conducted in R (v4.2.2). Covariate‐adjusted robust linear regression models, using the “rlm” function from the “MASS” package,^78^, ^79^ assessed the associations between each exposure (HDP subtype or BP trajectory) and the natural log‐transformed levels of each of the 90 widely detected EVP miRNAs. Participants without hypertension during pregnancy were the reference group for HDP subtypes, and the low BP trajectory was the reference for BP trajectory models. Associations between each exposure (HDP subtype or BP trajectory) and the 26 C14MC/C19MC miRNAs evaluated as secondary outcomes (ie, above versus below detection) were assessed using covariate‐adjusted logistic regression with the “glm function” from the “stats” package.^80^ In secondary analyses, we examined potential interactions between each predictor (HDP subtype or BP trajectory) and outcome, including infant sex, maternal prepregnancy BMI, and maternal perceived stress during pregnancy, modeled as cross‐product interaction terms in regression models. *P* values were adjusted for multiple testing using the “p.adjust” function from the “stats” package in R, specifying the Benjamini and Hochberg false discovery rate (FDR) procedure.^81^, ^82^ Nominal *P* values are also presented throughout the article both for transparency and potential future use in meta‐analyses.

Gene set enrichment analyses were conducted to identify potential biological pathways that may be altered in response to miRNAs related to elevated BP. First, we identified experimentally validated target genes of these miRNAs using miRTargetLink (v2.0), restricting to genes identified with “strong” evidence.^83^ Over‐ or underrepresentation of these target genes in Protein Analysis Through Evolutionary Relationships pathways was assessed using EnrichR.^84^ Pathways with a *P*_FDR_<0.05 were considered statistically significant.

## RESULTS

### Participant Characteristics

Overall, the analytic subset was comparable to excluded participants (Table1). However, participants in the analytic sample had a larger number of BP measures in their EMRs and a lower perceived stress score (Table1). The median age and BMI of participants in the study was 28.6 (interquartile range, 23.8–32.9) years and 27.8 (24.5–31.6) kg/m,^2^ respectively. Less than half (41.6%) of participants attended or graduated college. Participant characteristics are shown separately by BP trajectory and for participants with and without HDP in Tables S3 and S4. Most participants in the Low BP trajectory (85.3%), and over half (63.0%) in the Moderate BP trajectory had no HDP. In contrast, 81.5% of participants in the High BP class had some HDP subtype, predominantly preeclampsia.

### Latent Class Trajectory Analysis

**Figure 1:**
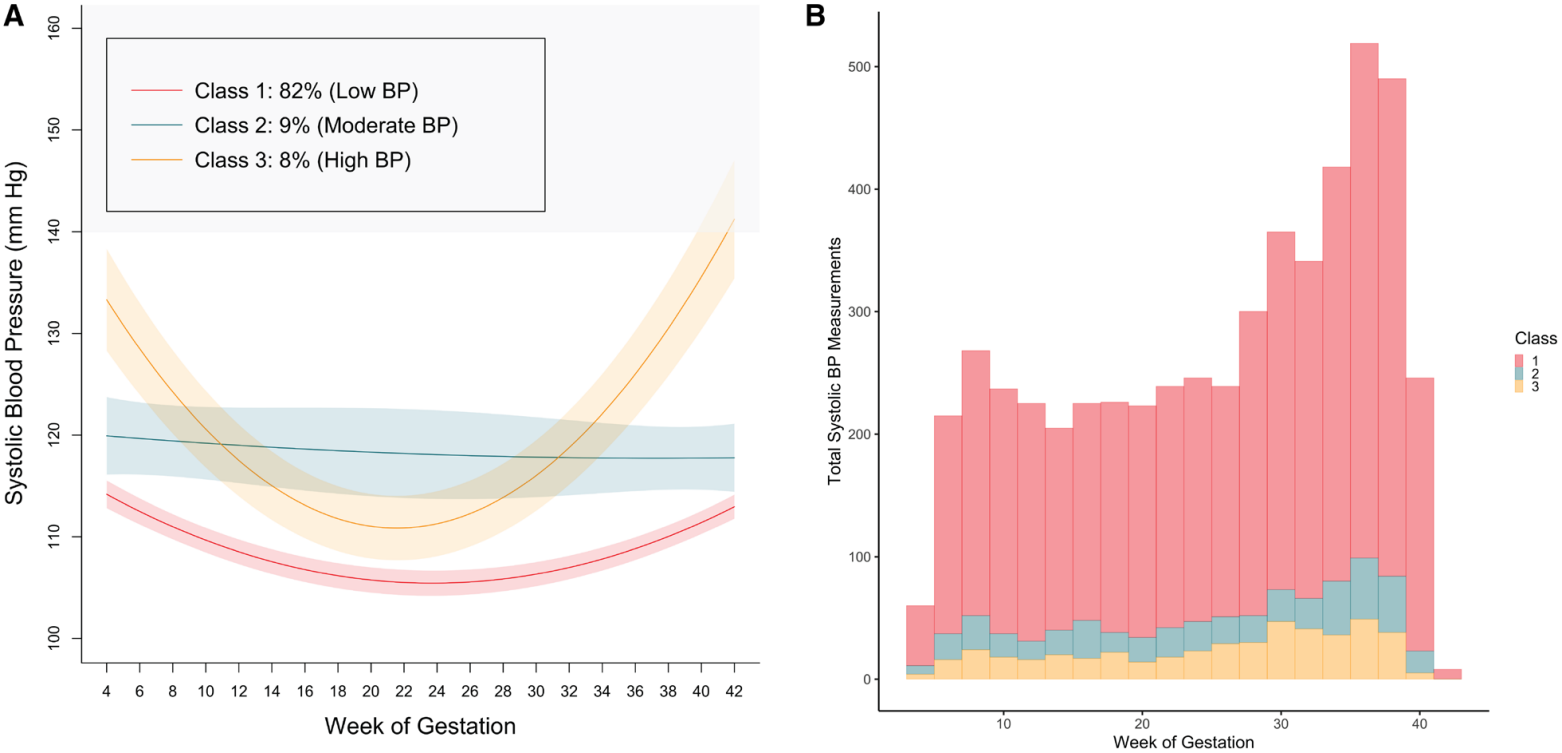
Systolic blood pressure trajectories. **A**, Latent class trajectory modeling identified 3 SBP trajectories. Median SBP across gestation, with 95% confidence bands, is plotted by trajectory. **B**, Distribution of SBP measurement collection across gestation by SBP trajectory. BP indicates blood pressure; and SBP, systolic blood pressure.

Three distinct latent classes of BP trajectories were identified (Figure 1): (1) Low BP, with largely normotensive BP measures that declined until ~20 weeks gestation, then rose steadily until delivery; (2) Moderate BP, with a median BP of ~120 mm Hg, remaining relatively consistent throughout gestation; and (3) High BP, starting with an elevated median BP (135 mm Hg) that declined to normotensive levels by 20 weeks gestation before rising again to an elevated level (median 140 mm Hg) at the end of pregnancy. The group names were assigned by the research team upon observation of the patterns and shape of each latent group. The average posterior probabilities for class membership was 0.89, 0.72, and 0.83, respectively.

In the LCTM model without a random slope component, the optimal number of classes increased from k=3 to k=4 (Table S1). This model separated the Moderate BP group into (1) a Moderate‐Low BP group with a noticeable dip in SBP at ~20 weeks gestation and (2) a Moderate‐High BP group that was similar to the k=3 Moderate BP class but featured an increase in SBP at ~20 weeks gestation, then declined until delivery (Figure S5). Participants in the k=4 Moderate‐Low BP group were predominantly classified as Low BP in the k=3 model, whereas participants in the k=4 Moderate‐High BP group were predominantly classified as Moderate BP in the k=3 model (Figure S6).

### Primary Analyses

**Figure 2:**
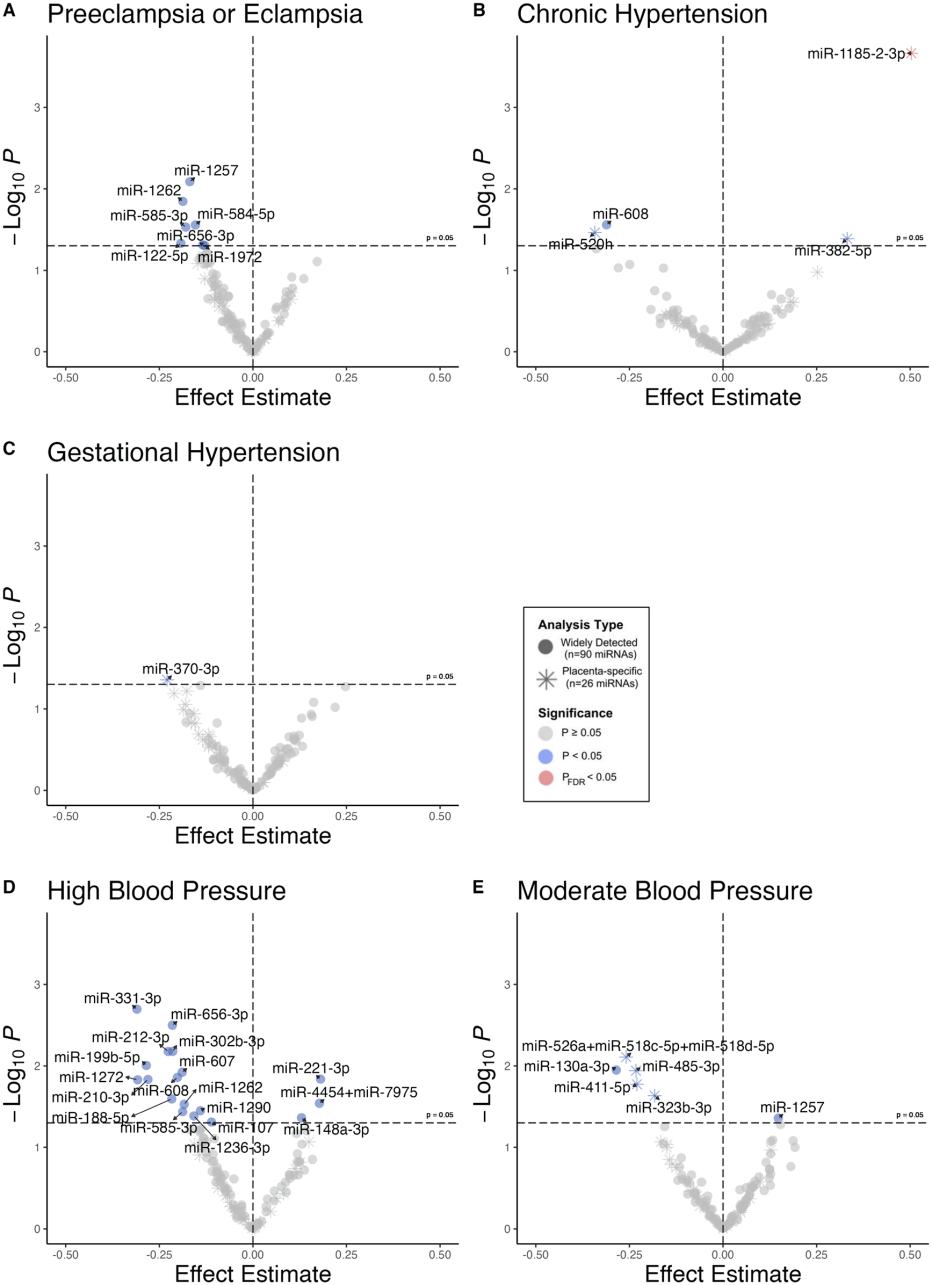
Volcano plots showing associations between HDP subtypes or BP trajectories and each miRNA. Volcano plots show effect estimates (*x* axis) and −log_10_ *P* values (*y* axis) from robust linear regression models (primary outcomes: widely detected miRNAs) and logistic regression models (secondary outcomes: low abundance C14MC/C19MC miRNAs). (A–C) Shows the association of HDP subtypes with miRNAs of interest. (D–E) Shows the association of High and Moderate BP trajectories and these miRNAs. All analyses were adjusted for maternal age, perceived stress during pregnancy, BMI, recruitment site, and timing of enrollment. BMI indicates body mass index; BP, blood pressure; C14MC, chromosome 14 microRNA cluster; C19MC, chromosome 19 microRNA cluster; FDR, false discovery rate; and HDP, hypertensive disorders of pregnancy.

Figure 2 presents associations between each (1) HDP subtype and (2) BP trajectory and EVP miRNA level. Participants with chronic hypertension were more likely to have detectable levels of 2 C14MC miRNAs: miR‐1185‐2‐3p and miR‐382‐5p (β, 0.50 [95% CI, 0.24–0.77] and 0.33 [95% CI, 0.01–0.65], respectively). The association for miR‐1185‐2‐3p remained statistically significant after multiple testing correction (*P*_FDR_<0.05). Participants with chronic hypertension were less likely to have detectable levels of miR‐520h, a C19MC miRNA, and lower levels of miR‐608, although these were not statistically significant (*P*_FDR_≥0.05). Participants with preeclampsia/eclampsia had lower levels of 7 widely detected miRNAs, including 1 C14MC miRNA: miR‐656‐3p (*P*<0.05), and participants with gestational hypertension had lower levels of 1 C14MC miRNA: miR‐370‐3p. However, these associations did not remain statistically significant after multiple testing correction (*P*_FDR_≥0.05).

**Figure 3:**
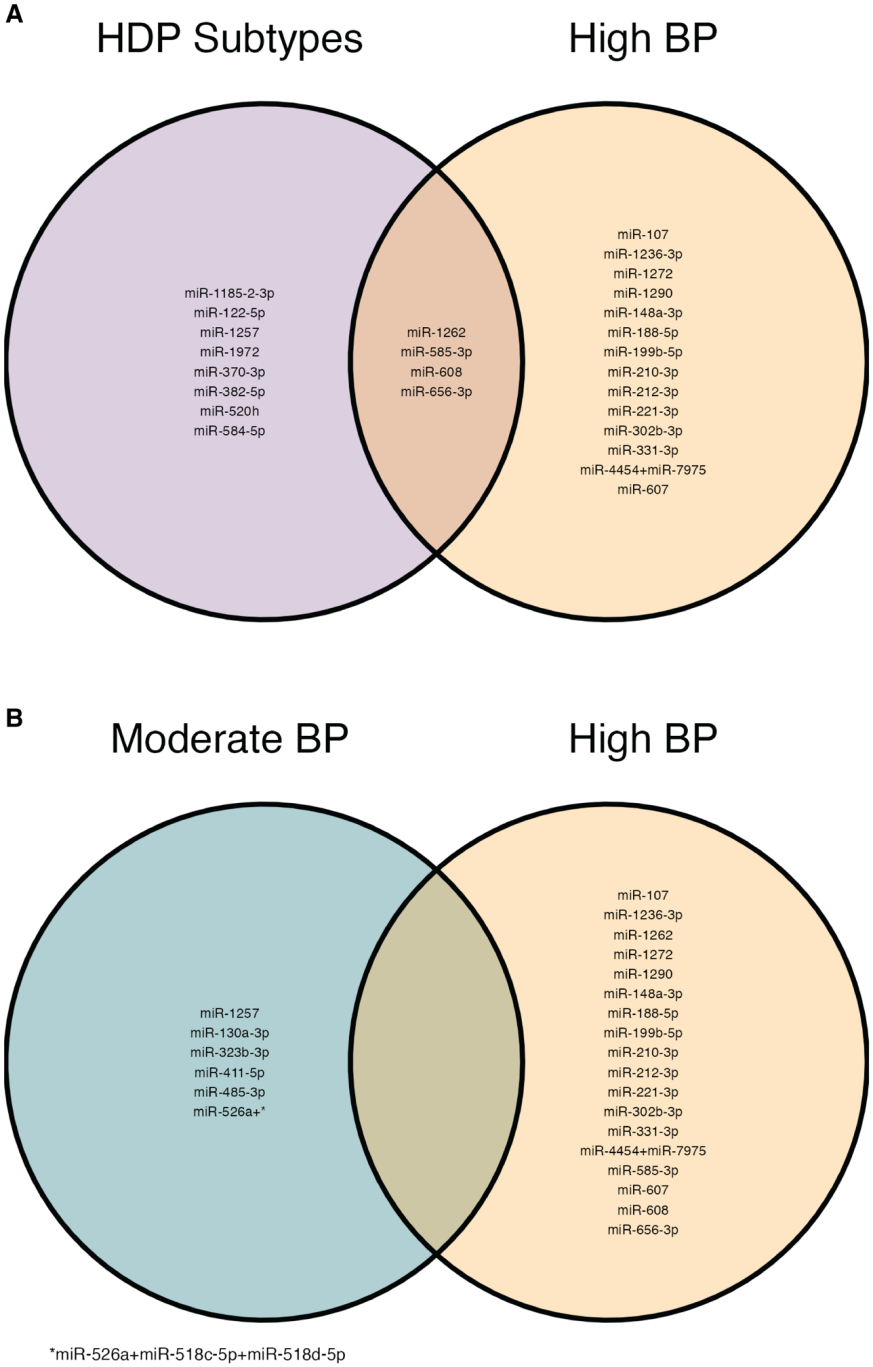
Venn diagrams showing overlap of miRNAs associated with HDP subtypes or BP trajectories (*P*<0.05). **A**, Compares results for any HDP subtype and the High BP trajectory. **B**, Compares the High and Moderate BP trajectories. All analyses were adjusted for maternal age, perceived stress during pregnancy, BMI, recruitment site, and timing of enrollment. BMI indicates body mass index; BP, blood pressure; and HDP, hypertensive disorders of pregnancy.

The High BP trajectory was associated with 18 widely detected miRNAs and the Moderate BP trajectory with 2 miRNAs. However, associations were not statistically significant after multiple testing correction (*P*_FDR_≥0.05). The majority of miRNAs were lower in relation to elevated BP. MiR‐656‐3p (from C14MC) had a lower abundance among participants in the High BP trajectory. (Table S5). An additional 4 placenta‐specific miRNAs that were assessed as secondary outcomes were less likely to be detected among participants in the Moderate BP trajectory (*P*<0.05). Four miRNAs were commonly associated with both the High BP trajectory and at least 1 HDP subtype (miR‐1262, miR‐585‐3p, miR‐608, and miR‐656‐3p), with 8 (66.7%) miRNAs uniquely associated with at least 1 HDP subtype and 14 (77.8%) miRNAs uniquely associated with the High BP class (Figure 3). There was no overlap between the miRNAs associated with the High BP trajectory versus Moderate BP trajectory.

A statistically significant interaction (*P*_FDR_=4.94×10^−4^) was identified between the Moderate BP trajectory and infant sex for miR‐30e‐5p. For male infants, the effect estimate for the Moderate BP trajectory was 0.26 (95% CI, −0.07 to 0.60) compared with −0.39 (95% CI, −0.59 to −0.18) for female infants. None of the other interactions assessed were statistically significant at *P*_FDR_<0.05.

### Gene Set Enrichment Analyses

**Figure 4:**
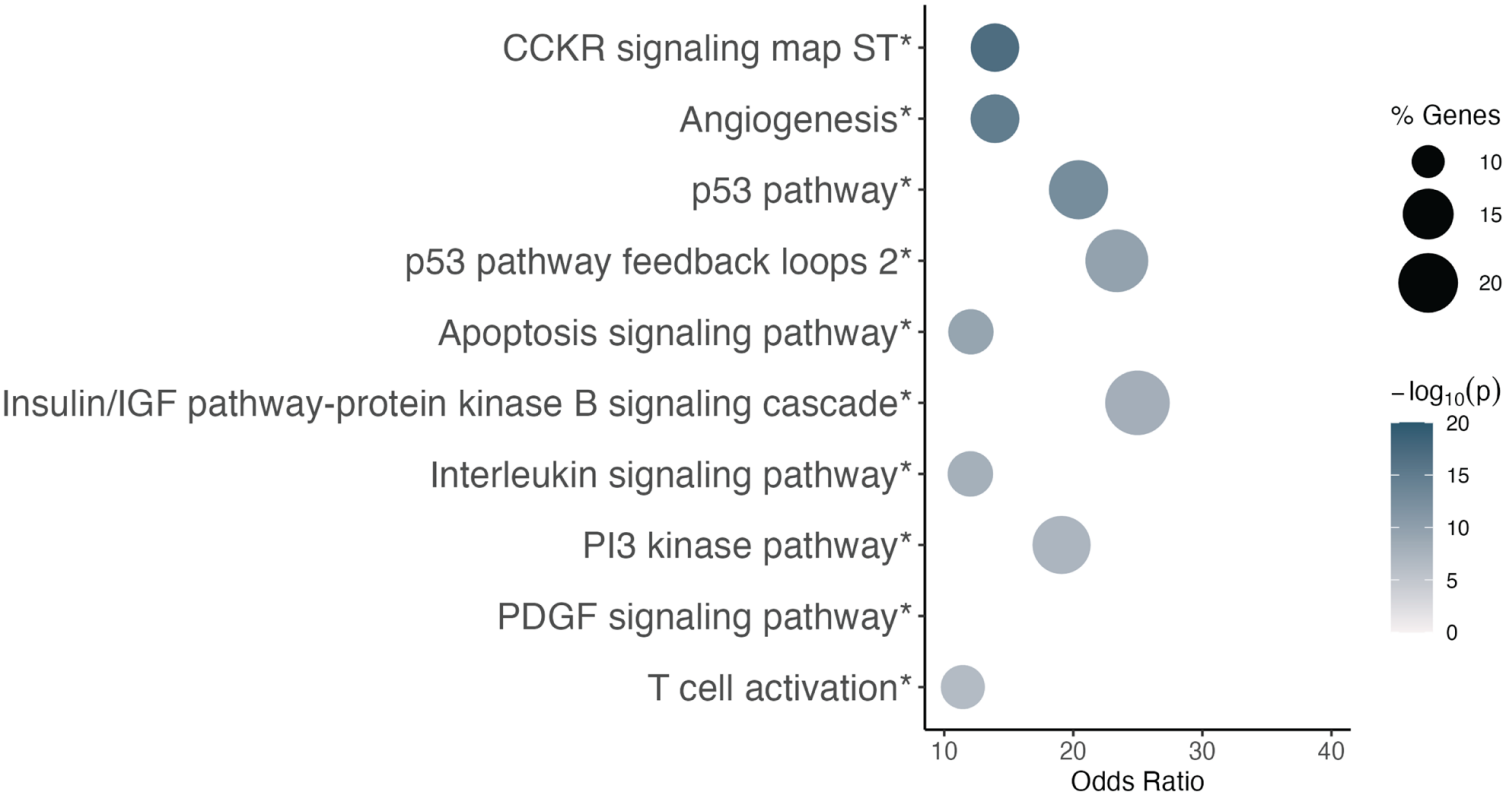
Gene set enrichment analysis results. Bubble plots showing the top 10 PANTHER pathways for miRNAs associated with the High BP trajectory. Bubble shade indicates the −log_10_ (*P* value) from Fisher's exact tests, with darker shades signifying smaller *P* values. Bubble size represents the percentage of genes in each pathway. The *x* axis shows the odds ratio from Fisher's exact tests. Asterisks following the pathway name signify statistical significance after multiple testing correction (*P*_FDR_<0.05). BP indicates blood pressure; IGF, insulin‐like growth factor; PANTHER, Protein Analysis Through Evolutionary Relationships; and PDGF, platelet‐derived growth factor.

Although no miRNAs were associated with any of the BP trajectories after multiple testing correction, a larger number of miRNAs were associated nominally with the High BP trajectory than would be expected by chance. We then focused our gene set enrichment analysis on the predicted target genes of miRNAs nominally (*P*<0.05) associated with the High BP trajectory (378 unique target genes of 18 miRNAs; Figure 4). Predicted gene targets were overrepresented in the CCKR signaling map, angiogenesis, the p53 pathway, the p53 pathway feedback loops 2, the apoptosis signaling pathway, the insulin/IGF (insulin‐like growth factor) pathway‐protein kinase B signaling cascade pathway, interleukin signaling, the PI3 kinase pathway, the PDGF (platelet‐derived growth factor) signaling pathway, and T‐cell activation at *P*_FDR_<0.05.

### Sensitivity Analyses

Results from sensitivity analyses were generally consistent with results from primary models (Table S6). However, the positive association between chronic hypertension and miR‐1185‐2‐3p (0.50; 0.24–0.77) was stronger (0.76; 0.40–1.13) after excluding participants with diabetes.

Results for the High BP group were similar across the k=4 and the k=3 models, but there was less overlap for the Moderate BP trajectory results (Figure S6). Overall, a much larger number of miRNAs were associated with the Moderate BP classes identified with the k=4 model (Figure S7). One probe (miR‐526a+miR‐518c‐5p+miR‐518d‐5p) covering 3 C19MC miRNAs was consistently lower in relation to the 2 Moderate BP classes identified using the k=4 model and with the 1 Moderate BP class identified using the k=3 model (Figure S6). In the k=4 model, this association was stronger in the Moderate‐High BP class (β, −0.46 [95% CI, −0.77 to −0.15]) compared with the Moderate‐Low BP class (β, −0.17 [95% CI, −0.27 to −0.06]), although both associations were statistically significant. Notably, in the k=4 analysis, 5 miRNA probes (all C14MC/C19MC miRNAs) were associated with 1 of the 2 Moderate BP classes at *P*_FDR_<0.05, none of which were associated with the Moderate BP class identified with the k=3 model.

## DISCUSSION

In the current study, we found that participants with chronic hypertension were more likely to have detectable circulating levels of miR‐1185‐2‐3p, a placenta‐specific miRNA, during pregnancy. This association remained statistically significant after multiple testing correction (*P*_FDR_<0.05). Although suggestive associations (*P*<0.05) were also identified for 36 additional miRNAs in relation to other HDP subtypes and BP trajectories, including a trajectory that reflected elevated BP in the subclinical range across gestation, these associations did not remain statistically significant after multiple testing correction. Many of the miRNAs that were nominally associated with elevated BP are highly expressed in the placenta and are predicted to target genes involved in pathways relevant to placental development and endothelial dysfunction.

The miRNA (miR‐1185‐2‐3p) that was elevated in participants with chronic hypertension is a C14MC miRNA,^33^, ^68^, ^69^ which is primarily expressed in the placenta and decreases across gestation.^33^ A prior study found that participants with rising levels of this miRNA across gestation had a higher risk of preterm delivery.^85^ Although C14MC miRNAs are primarily expressed in the placenta, they may also be detected in the blood of patients with various conditions, including cardiovascular disease.^86^, ^87^ Notably, a cross‐sectional study of nonpregnant adults reported higher circulating levels of miR‐1185 in participants with increased arterial stiffness and noted that this miRNA upregulates vascular cell adhesion molecule‐1 and E‐selectin, markers of endothelial dysfunction.^28^ In a separate study, the same group found that miR‐1185 induces apoptosis in endothelial cells and proposed that this miRNA could be a potential therapeutic target for the prevention of atherosclerosis.^22^ It is therefore plausible that this miRNA may contribute to the adverse effects of chronic hypertension during pregnancy, such as preterm birth and postpartum cardiovascular disease, and provide insight into susceptibility to these outcomes.^88^, ^89^

Most (83%) miRNAs suggestively associated (*P*<0.05) with either HDP subtypes or BP trajectories during pregnancy were lower among participants with elevated BP. Several of these miRNAs have cardioprotective roles (eg, miR‐30e‐3p and miR‐656‐3p) and many are expressed in the placenta (eg, miR‐370‐3p, miR‐526a+miR‐518c‐5p+miR+518d‐5p, and miR‐520h).^85^, ^90^ These inverse associations may reflect a downregulation in the expression or release of these from the placenta, possibly due to hypoxia, oxidative stress, and inflammation, which occur in response to elevated BP.^91^, ^92^ Interestingly, 14 of the miRNAs that were lower in participants with elevated BP were previously associated with a longer gestational duration in the same cohort (miR‐608, miR‐584‐5p, miR‐1972, miR‐656‐3p, miR‐331‐3p, miR‐1272, miR‐199b‐5p, miR‐210‐3p, miR‐212‐3p, miR‐302b‐3p, miR‐188‐5p, miR‐1236‐3p, miR‐1290, and miR‐107), and 2 of the miRNAs that were higher in participants with elevated BP were associated with a shorter gestational duration (miR‐4454 + miR‐7975, miR‐221‐3p).^50^ Collectively these results suggest that altered levels of these 16 miRNAs in relation to elevated BP could potentially contribute to a shorter gestational duration, a known consequence of elevated BP during pregnancy.^9^, ^93^

Notably, 76% of participants in the High BP class were also classified as having an HDP. Four miRNAs were lower among participants in the High BP class and with a HDP subtype: miR‐608, miR‐1262, miR‐585‐3p, and miR‐656‐3p. All of these miRNAs are involved in apoptosis, which contributes to endothelial dysfunction and activates inflammatory pathways.^94^, ^95^, ^96^, ^97^ Downregulation of miR‐608 has been associated with increased inflammation^98^ and elevated levels of acetylcholinesterase which is involved in hypertension development.^99^ MiR‐585‐3p is involved in cardiac cell differentiation and cardiomyocyte function,^100^ and reduced expression in endothelial cells has been associated with hypertension and coronary artery disease.^101^ MiR‐656‐3p is downregulated in response to hypoxia, which improves the viability and mobility of placental mesenchymal stem cells.^90^ Elevated BP can reduce blood flow to the placenta, causing hypoxia.^102^ Reductions in miR‐656‐3p in relation to elevated BP may therefore be an adaptive response to protect the developing fetus during hypoxic conditions.

Interestingly, no miRNAs overlapped between the High BP and Moderate BP trajectories. This could reflect distinct physiological responses to elevated BP in each range or the timing of elevated BP. For example, the Moderate BP trajectory did not demonstrate the classic midpregnancy dip in BP. In contrast, the High BP group had elevated BP levels in both early and late pregnancy but demonstrated a midpregnancy dip, with levels becoming comparable to the Low BP group during this window. Placental miRNA expression patterns change across gestation,^33^ thus their sensitivity to elevated BP may vary over time. For example, placental expression of miR‐199b‐5p is higher in the first trimester, and circulating levels of this miRNA were lower for participants in the High but not Moderate BP group.^33^

Despite prior studies reporting fetal sex differences for miRNA profiles in the placenta and maternal circulation during pregnancy,^103^ only 1 miRNA (miR‐30e‐3p) was differentially associated with elevated BP by fetal sex. Participants in the Moderate BP trajectory who carried a female fetus had lower circulating levels of miR‐30e‐3p in late pregnancy, which was not observed for participants carrying a male fetus. MiR‐30e‐3p is highly expressed in the placenta, especially in late pregnancy, and higher levels have been reported in male placentas, which may explain these sex differences.^103^ Cardioprotective roles have been reported for miR‐30e‐3p, with decreased expression in the myocardium contributing to cardiac dysfunction in rat models.^104^ Experimental studies have additionally found that miR‐30e‐3p protects against myocardial injury during hypoxia.^23^, ^105^, ^106^ However, higher expression of miR‐30e‐3p in cervical tissue has been associated with spontaneous preterm birth.^107^ Thus, although reductions in miR‐30e‐3p levels could be adaptive for the fetus, they may adversely affect maternal cardiovascular health.

In sensitivity analyses, we compared results from a trajectory analysis with 4 BP classes versus 3. Results for the High BP trajectory were similar across both models, likely due to substantial participant overlap, but were less consistent for the Moderate BP trajectories. Overall, more miRNAs were associated with the 2 Moderate BP trajectories from the k=4 model than with the single Moderate BP trajectory from the k=3 model. It is possible that the 2 Moderate BP trajectories identified with the k=4 model have distinct effects on circulating miRNAs, which would have been masked when these trajectories were collapsed in the k=3 model. Despite these differences, 1 probe reflecting 3 miRNAs (miR‐526a+miR‐518c‐5p+miR‐518d‐5p) was consistently lower among participants falling in any Moderate BP trajectory. In the k=4 model, this association was stronger for the Moderate‐High compared with Moderate‐Low BP trajectory. Higher circulating levels of miR‐526a (a C19MC miRNA) in late pregnancy have been associated with preeclampsia,^108^, ^109^ and upregulation of miR‐518d in placental tissue has been associated with low birth weight.^110^ Reduced levels of these miRNAs may therefore reflect adaptive responses to elevated BP during pregnancy.

Predicted target genes of miRNAs nominally associated with elevated BP were overrepresented in pathways associated with atherosclerosis, adverse pregnancy outcomes, and placental function.^111^, ^112^, ^113^ For example, target genes of miRNAs associated with the High BP trajectory were commonly overrepresented in angiogenesis, which is important for placental development.^114^, ^115^ Predicted target genes of miRNAs nominally associated with each of the different elevated BP measures were also overrepresented in the p53 feedback loop and p53 pathways, which are activated in response to placental hypoxia and involved in trophoblast differentiation and proliferation.^116^, ^117^, ^118^ These pathways are dysregulated in preeclampsia but have not been well studied for other HDP subtypes or elevated BP in the subclinical range.^119^, ^120^ The PI3 kinase pathway was commonly identified as a top pathway for miRNAs associated with the the High BP trajectory, is essential for trophoblast cell differentiation,^121^ and is altered in cardiometabolic diseases.^113^, ^122^, ^123^ Dysregulation of each of these pathways has been implicated in adverse maternal and child outcomes previously associated with elevated BP during pregnancy, such as preterm birth, placental abruption, and cardiovascular disease.^122^, ^123^, ^124^ Thus, alterations in these circulating miRNAs may represent a mechanism by which elevated BP during pregnancy contributes to adverse birth outcomes as well as long‐term cardiovascular outcomes in both mother and child.

There are some limitations worth acknowledging. First, although the NanoString nCounter platform covers a large number of miRNAs, it is not comprehensive. It is possible that additional miRNAs not profiled for this study may also be sensitive to elevated BP. Although this is one of the largest studies to evaluate elevated maternal BP during pregnancy in relation to circulating EVP miRNAs, analyses assessing the different HDP subtypes may have been underpowered. Despite the prospective design, we cannot rule out the possibility of reverse causality, as placental dysfunction can begin early in pregnancy and may be a cause (and a consequence) of hypertension.^125^, ^126^ Although a major strength is our focus on a population historically underrepresented in biomedical research, results may not be broadly generalizable, highlighting the need for additional research across other diverse populations. Additionally, while participants included in this study were largely comparable to the larger cohort, on average they had a larger number of BP measures during pregnancy (median=14 measures) compared with participants who were not included in the study (median=11 measures), which may reflect differences in access to care. Finally, it is important to acknowledge that many of the identified associations between elevated BP and circulating EVP miRNAs were not statistically significant after multiple testing correction. It will therefore be important to replicate these findings in a larger study.

Although LCTM offers valuable and innovative insights into complex longitudinal data, it is not without limitations. Model selection, particularly determining the optimal number of classes, can be sensitive to model parameters and structure, which increases the risk of overfitting or misclassification. Furthermore, LCTM assumes that class membership remains stable over time, which may not adequately reflect the dynamic nature of some longitudinal data. Interpretability may also pose challenges, especially when class distinctions are too homogeneous. Despite its limitations, LCTM remains a powerful, data‐driven tool. It can uncover latent subgroups that would be missed using simpler modeling strategies and may be indicative of subpopulations at higher risk for future cardiovascular disease. In support of this, prior work in the same cohort found that participants classified in the high and Moderate BP trajectories have an elevated risk of postpartum hypertension as compared with participants classified in the Low BP trajectory, indicating that even in the subclinical range elevated BP may adversely affect subsequent health.^17^ Results from the current study suggest that alterations in circulating EVP miRNAs could potentially contribute to some of these effects.

Key strengths of this research include the prospective design, the assessment of elevated BP in both the clinical and subclinical range, and the assessment of a large number of miRNAs in maternal circulating EVPs, including miRNAs known to be almost exclusively expressed in the placenta. Our assessment of maternal circulating EVP miRNA alterations in late pregnancy in relation to elevated BP is also a novel contribution, as prior studies have largely focused on maternal circulating EVP miRNAs measured in the first trimester due to an interest in identifying early biomarkers of pregnancy complications. By focusing on miRNAs in late pregnancy we were able to assess possible maternal and placental responses to elevated BP during pregnancy, which may explain some of the downstream consequences previously reported in this same population.

## CONCLUSIONS

In conclusion, our study suggests that a number of maternal circulating EVP miRNAs are sensitive to elevated BP, including in the subclinical range. These miRNAs regulate genes involved in pathways implicated in adverse birth outcomes and future cardiovascular risk. Further study of these miRNAs and the pathways that they regulate could aid in identifying minimally invasive biomarkers for individuals who may be more susceptible to the adverse effects of elevated maternal BP during pregnancy on health outcomes in the postpartum/postnatal period. Such biomarkers may inform targeted interventions that protect both maternal and child health.

## Sources of Funding

This research was supported by the National Institutes of Health [R01 MD011698, 4UH3OD023287‐03, R00 ES030400, P30 ES007048, P50 ES026086, R01 ES025145, P50 MD015705]; US Environmental Protection Agency [83615801‐0]; and the Burroughs Wellcome Fund [100000861] training grant at Dartmouth.

## Disclosures

None.

## Supporting information

Tables S1–S6
Figures S1–S7
