# Supplementary material for: Blood Pressure and Late Pregnancy Circulating miRNAs in the MADRES Study
Source: J Am Heart Assoc. 2025 Jun 11;14(12):e040416. doi: 10.1161/JAHA.124.040416 (PMC12229125; doi:10.1161/JAHA.124.040416)
Supplement: Supplementary file 1 — Tables S1–S6 Figures S1–S7 [file JAH3-14-e040416-s001.pdf]

Supplemental Materials

Table S1: Latent Class Trajectory Model Analysis Selection Criterium for the k=3 and k=4 models

A Criterium to Pick the Number of Classes (k)

Analysis	K	Log Likelihood	NPM	BIC	Class 1 %	Class 2 %	Class 3 %	Class 4 %	Class 5 %
Including Random Slope (k=3)*	1	-38777.69	10	77622.88	100%				
	2	-38705.8	15	77512.85	92%	8%			
	3***	-38689.7	20	77514.39	45%	48%	6%		
	4	-38681.16	25	77531.08	49%	43%	6%	7%	
	5	-38681.42	30	77565.34	12%	60%	20%	3%	5%
Not including random slope (k=4)	1	-36064.03	5	72451.57	100%				
	2	-36064.03	10	72194.65	87%	13%			
	3	-36013.25	15	72126.39	68%	26%	6%		
	4	-35986.08	20	72105.35	77%	15%	3%	5%	
	5	-35975.42	25	72117.32	14%	11%	67%	2%	5%

B Overall Model Diagnostics

Analysis	Model****	Entropy	BIC	% Class 1	% Class 2	% Class 3	% Class 4	APPA 1	APPA 2	APPA 3	APPA 4	OC 1	OC 2	OC 3	OC 4	MM 1	MM 2	MM 3	MM 4
Including Random Slope (k=3)	RT, CS	306.99	**77502.44**	82.32	9.48	8.2	NA	0.88	0.72	0.83	NA	2.44	15.61	43.6	NA	0.06	-0.04	-0.02	NA
	RT^2, CS	458.93	77514.39	45.08	48.48	6.44	NA	0.72	0.78	0.85	NA	3.71	3.4	59.06	NA	0.05	-0.03	-0.02	NA
	RT, CC	103.88	77537.54	1.99	92.74	5.27	NA	0.75	0.97	0.86	NA	88.41	3.06	99.33	NA	-0.01	0.02	-0.01	NA
	RT^2, CC	151.31	77537.55	90.4	4.33	5.27	NA	0.95	0.7	0.85	NA	2.75	36.09	88.07	NA	0.03	-0.02	-0.01	NA
Not including random slope (k=4)	RT, CS	472.401	**72056.1**	42%	47%	30%	80%	0.72	0.741	0.703	0.823	4.138	3.19	52.863	41.351	0.037	-0.002	-0.016	-0.019
	RT^2, CS	450.755	72072.7	63%	28%	40%	50%	0.782	0.706	0.74	0.872	2.87	5.296	39.104	100.259	0.075	-0.033	-0.032	-0.01
	RT, CC	96.042	72080.9	93%	1%	1%	5%	0.969	0.831	0.829	0.799	3.046	363.928	200.463	71.773	0.022	-0.003	-0.012	-0.006
	RT^2, CC	179.836	72076.8	89%	1%	4%	6%	0.935	0.792	0.725	0.834	2.483	169.868	39.832	75.137	0.042	-0.012	-0.026	-0.004

*Inclusion of a random slope allows the slope within each class to vary more. Not including a random slope in the model facilitates homogeneity within each class.

** Information about each column in Table A (Criterium to Pick the Number of Classes (k)) are included below

- NPM: Number of parameters per model
- BIC: Bayesian Inference Criterium, a lower value indicates a better fit

***Cells highlighted in yellow indicate which model was selected for the primary analysis. Text in red indicate the value is outside of recommended ranges.

***Information about each column in Table B (Overall Model Diagnostics) are included below

- Model: RT (random time), RT2 (random time squared), CS (class specific variance-covariance), and CC (class common variance-covariance) were uniquely combined for each model.
- Entropy: A higher entropy indicates more heterogeneous trajectories, a lower entropy indicates more homogeneous trajectories.
- BIC: Bayesian Inference Criterium, a lower value indicates a better fit.
- APPA: Average of the maximum posterior probability of assignments, a value >0.7 for each trajectory is ideal.
- OC: Odds of Correct Classification, a value >5 is ideal.
- MM: Mismatch, or the difference between the class proportions and membership. A value close to 0 is ideal.

Table S2: PCA Analysis

This table shows the association between potential confounders and Principal Component 1. P-values were calculated by the statistical test indicated in the row header for each section and P-values < 0.05 are highlighted in yellow.

	**Principal Component 1**
**Binary Variables (Wilcoxon Correlation)**	
Cohort Entry (Early or Late)	0.740986*
Infant Sex (Male or Female)	0.031901*
Blood Collection Time (Morning or Afternoon)	0.493049
HDP Diagnosis (yes or no)	0
**Categorical Variables (Kruskal-Wallis Correlation)**	
Marital Status (declined to answer, married, living together, single, divorced)	0.730604
Maternal Education (Less than 12th grade, 12th grade, some college, completed 4 years of college, some graduate training after college)	0.292105
Recruitment Site (Site 1, Site 2, Site 3, Site 4)	0.365823*
Smoking (smoked during pregnancy or not)	0.843875
HDP Category (PE/E, GESTATIONAL HYPERTENSION, CHTN with PE, and CHTN)	0.597285
**Continuous Variables (Spearman Correlation)**	
Maternal Age	0.460451*
Maternal BMI	0.194232
Stress (measured by the PSS)	0.179267*
Physical Activity (measured by the PPAQ)	0.013369
Depression (measured by the CES)	0.205455
Number of previous births (maternal order)	0.86968
Weeks of gestation at miRNA collection	0.431236

**Values with an asterisk were in the DAG minimal adjustment set or were study design covariates.*

Table S3: Participant Demographics by Trajectory Model (k=3 vs k=4)
This table shows key demographics for both the full MADRES cohort (n=1065) and the analytic subset (n=372), subset by the trajectories for both the k=3 and k=4 analyses. Values provided are either median (IQR) or n (%).

Full Cohort (n=1065)

			K = 3 Model				*K=4 Model*				
**Characteristic**		**Full Cohort (n=1065)**	**Low BP Trajectory (n=703)**	**Moderate BP Trajectory (n=81)**	**High BP Trajectory (n=70)**	**P-value**	**Low BP Trajectory (n=329)**	**Moderate-low BP Trajectory (n=424)**	**Moderate-high BP Trajectory (n=34)**	**High BP Trajectory (n=67)**	**Kruskal Wallis P-value**
Maternal age, years		27.98 (23.53-32.8)	27.97 (23.6-32.79)	26.59 (23.47-31.73)	30.2 (25.18-34.14)	0.04	28.1 (24.06-32.62)	27.86 (22.99-33.02)	26.62 (23.56-31.38)	29.71 (25.05-33.82)	0.10
Pre-pregnancy BMI, kg/m2		27.82 (23.84-32.74)	27.25 (23.39-31.37)	32.78 (28-38.4)	29.93 (26.06-34.21)	0.00	25.69 (22.6-29.06)	29.02 (24.67-33.47)	34.06 (30.13-38.84)	29.82 (25.78-34.18)	0.00
CES-D		8 (4-13)	8 (4.75-13)	7 (4-11)	9 (5-13.75)	0.55	8 (5-13)	8 (4-13)	7 (3-10.5)	9 (5-14)	0.49
PSS		13 (9-17)	13 (9-18)	10 (6-15)	13 (8.25-17)	0.01	13 (9-17)	13 (8-17)	9 (6-13.5)	13 (8.75-17.25)	0.03
PPAQ, MET-hours per week		245.2 (177.92-327.4)	244.93 (182.61-329.38)	251.66 (169.26-304.86)	257.49 (166.59-324.67)	0.87	246.06 (181.74-327.72)	241.5 (179.1-330.25)	256.34 (182.79-314.94)	257.49 (173.48-321.98)	0.99
Number of SBP measures during pregnancy		13 (9-16)	13 (9-16)	13 (11-17)	12 (9-16.75)	0.25	13 (9-15)	13 (9-16)	13 (10.25-16.75)	12 (9-16.5)	0.83
Gestational age at delivery, weeks		39.29 (38.14-40.14)	39.43 (38.43-40.29)	39.14 (38-40.14)	38.43 (37.04-39.11)	0.00	39.29 (38.43-40.14)	39.57 (38.43-40.29)	39.21 (38.07-40.25)	38.14 (37-39)	0.00
Gestational weight gain, kg		11.08 (6.06-15.62)	11.13 (6.3-15.53)	11.94 (5.87-15.6)	9.88 (6.61-14.47)	0.72	10.43 (6.3-14.03)	11.71 (6.06-16.46)	12.93 (8.36-15.92)	9.85 (6.22-14.23)	0.02
Birth order of child		2 (1-3)	2 (1-3)	2 (1-2)	2 (1-3)	0.14	2 (1-3)	2 (1-2)	2 (1-2)	2 (1-3)	0.00
Enrolled at >=20 weeks' gestation		274 (25.7%)	214 (30.4%)	23 (28.4%)	21 (30.0%)	0.93	89 (27.1%)	134 (31.6%)	14 (41.2%)	21 (31.3%)	0.27
Any diagnosed diabetes	Yes	304 (28.5%)	217 (30.9%)	34 (42.0%)	33 (47.1%)	0.01	100 (30.4%)	135 (31.8%)	19 (55.9%)	30 (44.8%)	0.01
	No	589 (55.3%)	467 (66.4%)	46 (56.8%)	36 (51.4%)		219 (66.6%)	279 (65.8%)	15 (44.1%)	36 (53.7%)	
	Missing	172 (16.2%)	19 (2.7%)	1 (1.2%)	1 (1.4%)		10 (3.0%)	10 (2.4%)	0 (0.0%)	1 (1.5%)	
Reported prenatal vitamin use	Yes	836 (78.5%)	587 (83.5%)	72 (88.9%)	60 (85.7%)	0.42	277 (84.2%)	352 (83.0%)	32 (94.1%)	58 (86.6%)	0.36
	No or missing	229 (21.5%)	116 (16.5%)	9 (11.1%)	10 (14.3%)		52 (15.8%)	72 (17.0%)	2 (5.9%)	9 (13.4%)	
Infant sex	Female	459 (43.1%)	359 (51.1%)	43 (53.1%)	29 (41.4%)	0.36	169 (51.4%)	212 (50.0%)	21 (61.8%)	29 (43.3%)	0.43
	Male	449 (42.2%)	341 (48.5%)	38 (46.9%)	39 (55.7%)		159 (48.3%)	210 (49.5%)	13 (38.2%)	36 (53.7%)	
	Missing	157 (14.7%)	3 (0.4%)	0 (0.0%)	2 (2.9%)		1 (0.3%)	2 (0.5%)	0 (0.0%)	2 (3.0%)	
HDP subtype	No HDP	711 (66.8%)	600 (85.3%)	51 (63.0%)	13 (18.6%)	0.00	305 (92.7%)	330 (77.8%)	18 (52.9%)	11 (16.4%)	0.00
	Preeclampsia / Eclampsia	106 (10.0%)	44 (6.3%)	12 (14.8%)	41 (58.6%)		8 (2.4%)	42 (9.9%)	7 (20.6%)	40 (59.7%)	
	Chronic Hypertension	26 (2.4%)	14 (2.0%)	6 (7.4%)	6 (8.6%)		4 (1.2%)	13 (3.1%)	3 (8.8%)	6 (9.0%)	
	Gestational Hypertension	62 (5.8%)	37 (5.3%)	12 (14.8%)	10 (14.3%)		8 (2.4%)	35 (8.3%)	6 (17.6%)	10 (14.9%)	
	Missing	160 (15.0%)	8 (1.1%)	0 (0.0%)	0 (0.0%)		4 (1.2%)	4 (0.9%)	0 (0.0%)	0 (0.0%)	
Maternal education	Less than 12th grade	245 (23.0%)	169 (24.0%)	13 (16.0%)	21 (30.0%)	0.03	86 (26.1%)	92 (21.7%)	7 (20.6%)	18 (26.9%)	0.32
	Completed 12th grade	322 (30.2%)	212 (30.2%)	22 (27.2%)	23 (32.9%)		100 (30.4%)	124 (29.2%)	11 (32.4%)	22 (32.8%)	
	Some college	257 (24.1%)	172 (24.5%)	28 (34.6%)	17 (24.3%)		79 (24.0%)	108 (25.5%)	12 (35.3%)	18 (26.9%)	
	Completed 4 years of college	108 (10.1%)	71 (10.1%)	12 (14.8%)	5 (7.1%)		32 (9.7%)	48 (11.3%)	3 (8.8%)	5 (7.5%)	
	Some graduate training	48 (4.5%)	40 (5.7%)	3 (3.7%)	1 (1.4%)		18 (5.5%)	24 (5.7%)	1 (2.9%)	1 (1.5%)	
	Missing	85 (8.0%)	39 (5.5%)	3 (3.7%)	3 (4.3%)		14 (4.3%)	28 (6.6%)	0 (0.0%)	3 (4.5%)	
Smoking	Did not smoke during pregnancy	845 (79.3%)	596 (84.8%)	71 (87.7%)	61 (87.1%)	0.52	285 (86.6%)	354 (83.5%)	31 (91.2%)	58 (86.6%)	0.85
	Smoked during pregnancy	20 (1.9%)	12 (1.7%)	1 (1.2%)	0 (0.0%)		6 (1.8%)	5 (1.2%)	1 (2.9%)	1 (1.5%)	
	Missing	200 (18.8%)	95 (13.5%)	9 (11.1%)	9 (12.9%)		38 (11.6%)	65 (15.3%)	2 (5.9%)	8 (11.9%)	

Analytic Subset (n=372)

		K = 3 Model				*K=4 Model*				
**Characteristic**	**Analytic Subset (n=372)**	**Low BP Trajectory (n=308)**	**Moderate BP Trajectory (n=31)**	**High BP Trajectory (n=33)**	**P-value**	**Low BP Trajectory (n=153)**	**Moderate-low BP Trajectory (n=177)**	**Moderate-high BP Trajectory (n=11)**	**High BP Trajectory (n=31)**	**P-value**
Maternal age, years	28.59 (23.79-32.86)	28.59 (23.77-32.86)	25.9 (23.4-30.91)	30.89 (25.58-34.11)	0.14	28.81 (23.98-32.56)	28.07 (23-33.26)	29.13 (24.82-31.85)	29.71 (25.24-32.56)	0.74

Pre-pregnancy BMI, kg/m2		27.83 (24.53-31.64)	27.21 (24.24-31.13)	32.04 (28.23-35.66)	29.15 (25.85-33.2)	0.00	26.18 (23.28-29.61)	28.47 (25.25-33.12)	33.47 (31.34-37.06)	29.38 (25.78-33.21)	0.00
CES-D		8 (4-13)	8 (4-13)	7 (4-9)	7 (4-10)	0.34	7 (5-13)	8 (4-13)	5 (2-8)	8 (4-11)	0.27
PSS		12 (7-17)	12 (7-17)	9 (7-13)	13 (8-18)	0.05	12 (8-17)	12 (7-16)	8 (6.5-9.5)	13 (8.5-18.5)	0.16
PPAQ, MET-hours per week		250.79 (187.92-331.49)	252.04 (190.92-332.16)	248.75 (155.64-361.72)	235.75 (166.55-306.65)	0.64	254.32 (191.32-328.47)	240.98 (188.01-336.89)	263.92 (203.3-382.68)	246.1 (180.07-306.56)	0.67
Number of SBP measures during pregnancy		14 (11-17)	14 (11.75-17)	14 (11.5-18)	13 (9-18)	0.66	14 (11-17)	14 (12-17)	13 (12.5-17.5)	15 (9.5-17.5)	0.88
Gestational age at delivery, weeks		39.29 (38.29-40.14)	39.43 (38.57-40.29)	39.14 (37.93-40)	38.43 (37.29-39)	0.00	39.43 (38.71-40.14)	39.29 (38.29-40.29)	39.57 (38.5-40.21)	38 (37.14-39)	0.00
Gestational weight gain, kg		10.7 (5.94-15.26)	10.62 (5.97-14.91)	13.1 (8.16-16.79)	9.98 (4.57-19.26)	0.45	10.52 (5.94-14.23)	11.5 (7.09-16.45)	12.54 (6.3-15.5)	9.85 (4.55-19)	0.29
Birth order of child		2 (1-3)	2 (1-3)	2 (1-2)	2 (2-3)	0.26	2 (2-3)	2 (1-3)	2 (2-2)	2 (1.25-3)	0.03
Enrolled at >=20 weeks' gestation		95 (25.5%)	78 (25.3%)	6 (19.4%)	11 (33.3%)	0.43	34 (22.2%)	46 (26.0%)	4 (36.4%)	11 (35.5%)	0.36
Any diagnosed diabetes	Yes	131 (35.2%)	106 (34.4%)	10 (32.3%)	15 (45.5%)	0.42	51 (33.3%)	62 (35.0%)	5 (45.5%)	13 (41.9%)	0.72
	No	241 (64.8%)	202 (65.6%)	21 (67.7%)	18 (54.5%)		102 (66.7%)	115 (65.0%)	6 (54.5%)	18 (58.1%)	
	Missing	0 (0.0%)	0 (0.0%)	0 (0.0%)	0 (0.0%)		0 (0.0%)	0 (0.0%)	0 (0.0%)	0 (0.0%)	
Reported prenatal vitamin use	Yes	363 (97.6%)	300 (97.4%)	31 (100.0%)	32 (97.0%)	0.65	148 (96.7%)	174 (98.3%)	11 (100.0%)	30 (96.8%)	0.75
	No or missing	9 (2.4%)	8 (2.6%)	0 (0.0%)	1 (3.0%)		5 (3.3%)	3 (1.7%)	0 (0.0%)	1 (3.2%)	
Infant sex	Female	178 (47.8%)	156 (50.6%)	13 (41.9%)	9 (27.3%)	0.03	74 (48.4%)	88 (49.7%)	6 (54.5%)	10 (32.3%)	0.32
	Male	194 (52.2%)	152 (49.4%)	18 (58.1%)	24 (72.7%)		79 (51.6%)	89 (50.3%)	5 (45.5%)	21 (67.7%)	
	Missing	0 (0.0%)	0 (0.0%)	0 (0.0%)	0 (0.0%)		0 (0.0%)	0 (0.0%)	0 (0.0%)	0 (0.0%)	
HDP subtype	No HDP	300 (80.6%)	275 (89.3%)	17 (54.8%)	8 (24.2%)	0.00	146 (95.4%)	143 (80.8%)	4 (36.4%)	7 (22.6%)	0.00
	Preeclampsia / Eclampsia	41 (11.0%)	20 (6.5%)	6 (19.4%)	15 (45.5%)		4 (2.6%)	19 (10.7%)	4 (36.4%)	14 (45.2%)	
	Chronic Hypertension	10 (2.7%)	3 (1.0%)	3 (9.7%)	4 (12.1%)		1 (0.7%)	4 (2.3%)	1 (9.1%)	4 (12.9%)	
	Gestational Hypertension	21 (5.6%)	10 (3.2%)	5 (16.1%)	6 (18.2%)		2 (1.3%)	11 (6.2%)	2 (18.2%)	6 (19.4%)	
	Missing	0 (0.0%)	0 (0.0%)	0 (0.0%)	0 (0.0%)		0 (0.0%)	0 (0.0%)	0 (0.0%)	0 (0.0%)	
Maternal education	Less than 12th grade	99 (26.6%)	80 (26.0%)	6 (19.4%)	13 (39.4%)	0.09	39 (25.5%)	46 (26.0%)	3 (27.3%)	11 (35.5%)	0.38
	Completed 12th grade	114 (30.6%)	95 (30.8%)	9 (29.0%)	10 (30.3%)		50 (32.7%)	50 (28.2%)	5 (45.5%)	9 (29.0%)	
	Some college	105 (28.2%)	87 (28.2%)	10 (32.3%)	8 (24.2%)		42 (27.5%)	51 (28.8%)	3 (27.3%)	9 (29.0%)	
	Completed 4 years of college	38 (10.2%)	31 (10.1%)	5 (16.1%)	2 (6.1%)		15 (9.8%)	21 (11.9%)	0 (0.0%)	2 (6.5%)	
	Some graduate training	12 (3.2%)	11 (3.6%)	1 (3.2%)	0 (0.0%)		4 (2.6%)	8 (4.5%)	0 (0.0%)	0 (0.0%)	
	Missing	4 (1.1%)	4 (1.3%)	0 (0.0%)	0 (0.0%)		3 (2.0%)	1 (0.6%)	0 (0.0%)	0 (0.0%)	
Smoking	Did not smoke during pregnancy	365 (98.1%)	301 (97.7%)	31 (100.0%)	33 (100.0%)	0.48	149 (97.4%)	175 (98.9%)	11 (100.0%)	30 (96.8%)	0.68
	Smoked during pregnancy	7 (1.9%)	7 (2.3%)	0 (0.0%)	0 (0.0%)		4 (2.6%)	2 (1.1%)	0 (0.0%)	1 (3.2%)	
	Missing	0 (0.0%)	0 (0.0%)	0 (0.0%)	0 (0.0%)		0 (0.0%)	0 (0.0%)	0 (0.0%)	0 (0.0%)	

Table S4: Participant Demographics by Hypertensive Disorder of Pregnancy

Full Cohort (n=1065)

Characteristic		Full Cohort (n=1065)	Normal (n=711)	CHTN (n=26)	PE (n=106)	PIH (n=62)	Missing (n=160)	Kruskal Wallis P-value
Maternal age, years		27.98 (23.53-32.8)	27.85 (23.63-32.58)	28.05 (23.94-32.22)	29.42 (24.03-34.03)	28.26 (22.86-32.46)	27.6 (22.78-33.31)	0.04
Pre-pregnancy BMI, kg/m2		27.82 (23.84-32.74)	27.22 (23.45-31.3)	32.85 (24.06-37.28)	31.5 (27-35.34)	30.09 (24.35-35.5)	28.1 (24.58-34.11)	0.00
CES-D		8 (4-13)	7 (4-13)	8.5 (4.25-11.5)	8.5 (5-13)	9 (5-14.5)	7 (4-14.25)	0.55
PSS		13 (9-17)	13 (9-17)	12 (9-13.75)	12 (8-16)	14 (9-19)	13 (10.75-14.5)	0.01
PPAQ, MET-hours per week		245.2 (177.92-327.4)	245.54 (182.29-325.87)	310.6 (225.94-343.4)	244.5 (161.22-335)	222.5 (157.63-324.06)	187.49 (134.62-252.96)	0.87
Number of SBP measures during pregnancy		13 (9-16)	13 (9-16)	13 (8.25-16.75)	13 (10-18)	12 (8-15)	3 (2.75-4)	0.25
Gestational age at delivery, weeks		39.29 (38.14-40.14)	39.43 (38.43-40.29)	39 (37.86-39.57)	38.64 (37.14-40)	38.93 (37.61-40)	39.43 (38.64-40)	0.00
Gestational weight gain, kg		11.08 (6.06-15.62)	11.1 (6.52-15.46)	8.32 (0.25-13.29)	11.79 (7.22-16.78)	10.77 (5.87-16.87)	0.44 (0.3-2.32)	0.72
Birth order of child		2 (1-3)	2 (1-3)	2 (1-3)	1.5 (1-3)	1 (1-2)	2 (1-3)	0.14
Enrolled at >=20 weeks' gestation		274 (25.7%)	209 (29.4%)	8 (30.8%)	34 (32.1%)	22 (35.5%)	1 (0.6%)	0.93
Any diagnosed diabetes	Yes	304 (28.5%)	222 (31.2%)	10 (38.5%)	46 (43.4%)	25 (40.3%)	1 (0.6%)	0.01
	No	589 (55.3%)	476 (66.9%)	14 (53.8%)	58 (54.7%)	35 (56.5%)	6 (3.8%)	
	Missing	172 (16.2%)	13 (1.8%)	2 (7.7%)	2 (1.9%)	2 (3.2%)	153 (95.6%)	
Reported prenatal vitamin use	Yes	836 (78.5%)	609 (85.7%)	19 (73.1%)	88 (83.0%)	54 (87.1%)	66 (41.2%)	0.42
	No or missing	229 (21.5%)	102 (14.3%)	7 (26.9%)	18 (17.0%)	8 (12.9%)	94 (58.8%)	
Infant sex	Female	459 (43.1%)	360 (50.6%)	14 (53.8%)	50 (47.2%)	31 (50.0%)	4 (2.5%)	0.36
	Male	449 (42.2%)	345 (48.5%)	12 (46.2%)	55 (51.9%)	30 (48.4%)	7 (4.4%)	
	Missing	157 (14.7%)	6 (0.8%)	0 (0.0%)	1 (0.9%)	1 (1.6%)	149 (93.1%)	
Maternal education	Less than 12th grade	245 (23.0%)	167 (23.5%)	9 (34.6%)	25 (23.6%)	14 (22.6%)	30 (18.8%)	0.03
	Completed 12th grade	322 (30.2%)	216 (30.4%)	6 (23.1%)	37 (34.9%)	14 (22.6%)	49 (30.6%)	
	Some college	257 (24.1%)	176 (24.8%)	8 (30.8%)	24 (22.6%)	20 (32.3%)	29 (18.1%)	
	Completed 4 years of college	108 (10.1%)	78 (11.0%)	2 (7.7%)	9 (8.5%)	7 (11.3%)	12 (7.5%)	
	Some graduate training	48 (4.5%)	39 (5.5%)	0 (0.0%)	3 (2.8%)	4 (6.5%)	2 (1.2%)	
	Missing	85 (8.0%)	35 (4.9%)	1 (3.8%)	8 (7.5%)	3 (4.8%)	38 (23.8%)	
Smoking	Did not smoke during pregnancy	845 (79.3%)	615 (86.5%)	18 (69.2%)	91 (85.8%)	54 (87.1%)	67 (41.9%)	0.52
	Smoked during pregnancy	20 (1.9%)	12 (1.7%)	2 (7.7%)	0 (0.0%)	0 (0.0%)	6 (3.8%)	
	Missing	200 (18.8%)	84 (11.8%)	6 (23.1%)	15 (14.2%)	8 (12.9%)	87 (54.4%)	

Analytic Subset (n=372)

Characteristic		Analytic Subset (n=372)	Normal (n=300)	CHTN (n=10)	PE (n=41)	PIH (n=21)	Kruskal Wallis P-value
Maternal age, years		28.59 (23.79-32.86)	28.21 (23.7-32.64)	29.92 (28.04-32.05)	31.16 (23.85-34.57)	30.22 (25.58-32.56)	0.14
Pre-pregnancy BMI, kg/m2		27.83 (24.53-31.64)	27.3 (24.24-31.07)	34.02 (25.45-38.8)	29.43 (26.68-32.17)	32.66 (25.85-36.52)	0.00
CES-D		8 (4-13)	8 (4-13)	7.5 (4-9)	7 (4-10)	9 (6-16)	0.34
PSS		12 (7-17)	12 (7-17)	11 (9-13)	10 (7-15)	13 (7-19)	0.05
PPAQ, MET-hours per week		250.79 (187.92-331.49)	252.7 (192.02-329.58)	262.11 (210.46-312.66)	235.75 (162.57-331.95)	257.62 (185.88-345.7)	0.64
Number of SBP measures during pregnancy		14 (11-17)	14 (11-17)	14.5 (12.25-16.75)	15 (12-18)	16 (11-19)	0.66
Gestational age at delivery, weeks		39.29 (38.29-40.14)	39.43 (38.57-40.29)	38.93 (37.79-39.57)	38.71 (37.57-39.71)	38.86 (37.71-39.29)	0.00
Gestational weight gain, kg		10.7 (5.94-15.26)	10.48 (5.99-14.69)	10.12 (1.14-17.32)	11.56 (8.17-19.28)	9.87 (4.83-15.23)	0.45
Birth order of child		2 (1-3)	2 (1-3)	2 (2-3.75)	2 (1-3)	2 (1-2.5)	0.26
Enrolled at >=20 weeks' gestation		95 (25.5%)	71 (23.7%)	4 (40.0%)	14 (34.1%)	6 (28.6%)	0.43
Any diagnosed diabetes	Yes	131 (35.2%)	97 (32.3%)	5 (50.0%)	19 (46.3%)	10 (47.6%)	0.42
	No	241 (64.8%)	203 (67.7%)	5 (50.0%)	22 (53.7%)	11 (52.4%)	
	Missing	0 (0.0%)	0 (0.0%)	0 (0.0%)	0 (0.0%)	0 (0.0%)	
Reported prenatal vitamin use	Yes	363 (97.6%)	294 (98.0%)	9 (90.0%)	39 (95.1%)	21 (100.0%)	0.65
	No or missing	9 (2.4%)	6 (2.0%)	1 (10.0%)	2 (4.9%)	0 (0.0%)	
Infant sex	Female	178 (47.8%)	146 (48.7%)	4 (40.0%)	18 (43.9%)	10 (47.6%)	0.03
	Male	194 (52.2%)	154 (51.3%)	6 (60.0%)	23 (56.1%)	11 (52.4%)	
	Missing	0 (0.0%)	0 (0.0%)	0 (0.0%)	0 (0.0%)	0 (0.0%)	
Maternal education	Less than 12th grade	99 (26.6%)	76 (25.3%)	4 (40.0%)	14 (34.1%)	5 (23.8%)	0.09
	Completed 12th grade	114 (30.6%)	92 (30.7%)	4 (40.0%)	14 (34.1%)	4 (19.0%)	
	Some college	105 (28.2%)	86 (28.7%)	1 (10.0%)	9 (22.0%)	9 (42.9%)	
	Completed 4 years of college	38 (10.2%)	32 (10.7%)	1 (10.0%)	3 (7.3%)	2 (9.5%)	
	Some graduate training	12 (3.2%)	10 (3.3%)	0 (0.0%)	1 (2.4%)	1 (4.8%)	
	Missing	4 (1.1%)	4 (1.3%)	0 (0.0%)	0 (0.0%)	0 (0.0%)	

Smoking	Did not smoke during pregnancy	365 (98.1%)	293 (97.7%)	10 (100.0%)	41 (100.0%)	21 (100.0%)	0.48
	Smoked during pregnancy	7 (1.9%)	7 (2.3%)	0 (0.0%)	0 (0.0%)	0 (0.0%)	
	Missing	0 (0.0%)	0 (0.0%)	0 (0.0%)	0 (0.0%)	0 (0.0%)	

Table S5: Associations between Hypertensive Subtypes and Blood Pressure Trajectories and circulating EVP miRNAs (P< 0.05)
Bolded rows highlight miRNAs with PFDR < 0.05. All analyses were adjusted for maternal age, perceived stress during pregnancy, BMI, recruitment site, and timing of enrollment.

Analysis	Predictor*	Outcome	Placenta specific?	Primary or Secondary Outcome**	Effect Estimate (95% confidence interval)	P-value	FDR P-value
**Hypertensive subtypes**	Chronic Hypertension	miR-608	No	Primary	-0.31 (-0.59, -0.03)	0.028	0.915
	Preeclampsia/Eclampsia	miR-1257	No	Primary	-0.17 (-0.29, -0.05)	0.008	0.915
	Preeclampsia/Eclampsia	miR-1262	No	Primary	-0.19 (-0.34, -0.04)	0.014	0.915
	Preeclampsia/Eclampsia	miR-584-5p	No	Primary	-0.15 (-0.29, -0.02)	0.028	0.915
	Preeclampsia/Eclampsia	miR-585-3p	No	Primary	-0.18 (-0.34, -0.02)	0.029	0.915
	Preeclampsia/Eclampsia	miR-122-5p	No	Primary	-0.19 (-0.38, -0.01)	0.047	0.915
	Preeclampsia/Eclampsia	miR-1972	No	Primary	-0.13 (-0.26, 0)	0.049	0.915
	Preeclampsia/Eclampsia	miR-656-3p	Yes	Primary	-0.13 (-0.26, -0.01)	0.050	0.915
	**Chronic Hypertension**	**miR-1185-2-3p**	**Yes**	**Secondary**	**0.50 (0.24, 0.77)**	**2.16E-04**	**0.017**
	Chronic Hypertension	miR-520h	Yes	Secondary	-0.34 (-0.66, -0.03)	0.034	0.715
	Chronic Hypertension	miR-382-5p	Yes	Secondary	0.33 (0.01, 0.65)	0.041	0.715
	Gestational Hypertension	miR-370-3p	Yes	Secondary	-0.23 (-0.45, -0.01)	0.044	0.715
**K = 3 Analysis**	High BP	miR-331-3p	No	Primary	-0.31 (-0.51, -0.11)	0.002	0.242
	High BP	miR-656-3p	Yes	Primary	-0.22 (-0.36, -0.07)	0.003	0.242
	High BP	miR-1272	No	Primary	-0.31 (-0.56, -0.06)	0.007	0.242
	High BP	miR-199b-5p	No	Primary	-0.28 (-0.5, -0.07)	0.007	0.242
	High BP	miR-210-3p	No	Primary	-0.28 (-0.5, -0.06)	0.010	0.242
	High BP	miR-212-3p	No	Primary	-0.23 (-0.39, -0.06)	0.012	0.242
	High BP	miR-221-3p	No	Primary	0.18 (0.04, 0.33)	0.014	0.242
	High BP	miR-302b-3p	No	Primary	-0.21 (-0.37, -0.06)	0.015	0.242
	High BP	miR-607	No	Primary	-0.19 (-0.34, -0.04)	0.015	0.242
	High BP	miR-608	No	Primary	-0.20 (-0.36, -0.04)	0.015	0.242
	High BP	miR-1262	No	Primary	-0.18 (-0.35, -0.02)	0.025	0.381
	High BP	miR-188-5p	No	Primary	-0.22 (-0.41, -0.03)	0.029	0.381
	High BP	miR-4454+miR-7975	No	Primary	0.18 (0.02, 0.34)	0.030	0.381
	High BP	miR-1236-3p	No	Primary	-0.16 (-0.31, -0.01)	0.035	0.410
	High BP	miR-1290	No	Primary	-0.14 (-0.27, -0.01)	0.036	0.410
	High BP	miR-148a-3p	No	Primary	0.13 (0, 0.26)	0.041	0.415
	High BP	miR-585-3p	No	Primary	-0.19 (-0.36, -0.01)	0.043	0.415

	High BP	miR-107	No	Primary	-0.11 (-0.22, 0)	0.049	0.430
	Moderate BP	miR-130a-3p	No	Primary	-0.29 (-0.5, -0.07)	0.011	0.242
	Moderate BP	miR-1257	No	Primary	0.15 (0.01, 0.29)	0.044	0.415
	Moderate BP	hsa-miR-526a+hsa-miR-518c-5p+hsa-miR-518d-5p	Yes	Secondary	-0.26 (-0.45, -0.07)	0.008	0.291
	Moderate BP	hsa-miR-485-3p	Yes	Secondary	-0.23 (-0.41, -0.05)	0.011	0.291
	Moderate BP	hsa-miR-411-5p	Yes	Secondary	-0.23 (-0.42, -0.04)	0.017	0.291
	Moderate BP	hsa-miR-323b-3p	Yes	Secondary	-0.18 (-0.34, -0.03)	0.023	0.302
**K=4 Analysis**	High BP	miR-331-3p	No	Primary	-0.34 (-0.55, -0.14)	0.001	0.137
	High BP	miR-607	No	Primary	-0.23 (-0.39, -0.07)	0.003	0.137
	High BP	miR-656-3p	Yes	Primary	-0.24 (-0.39, -0.08)	0.004	0.151
	High BP	miR-1262	No	Primary	-0.25 (-0.43, -0.07)	0.005	0.151
	High BP	miR-210-3p	No	Primary	-0.32 (-0.54, -0.09)	0.006	0.151
	High BP	miR-212-3p	No	Primary	-0.23 (-0.41, -0.06)	0.006	0.151
	High BP	miR-221-3p	No	Primary	0.20 (0.04, 0.35)	0.007	0.155
	High BP	miR-302b-3p	No	Primary	-0.23 (-0.4, -0.06)	0.010	0.163
	High BP	miR-4454+miR-7975	No	Primary	0.22 (0.05, 0.39)	0.012	0.179
	High BP	miR-585-3p	No	Primary	-0.24 (-0.43, -0.05)	0.013	0.179
	High BP	miR-608	No	Primary	-0.24 (-0.41, -0.07)	0.013	0.179
	High BP	miR-1236-3p	No	Primary	-0.20 (-0.36, -0.04)	0.015	0.179
	High BP	miR-1272	No	Primary	-0.33 (-0.59, -0.06)	0.015	0.179
	High BP	miR-1972	No	Primary	-0.18 (-0.33, -0.04)	0.016	0.179
	High BP	miR-199b-5p	No	Primary	-0.28 (-0.5, -0.05)	0.017	0.179
	High BP	miR-548ar-3p	No	Primary	-0.19 (-0.36, -0.02)	0.024	0.227
	High BP	miR-126-3p	No	Primary	0.18 (0.02, 0.35)	0.026	0.231
	High BP	miR-188-5p	No	Primary	-0.22 (-0.43, -0.02)	0.028	0.242
	High BP	miR-299-5p	Yes	Primary	-0.18 (-0.34, -0.02)	0.033	0.267
	High BP	miR-29b-3p	No	Primary	0.13 (0, 0.26)	0.039	0.299
	High BP	miR-937-3p	No	Primary	-0.20 (-0.39, -0.01)	0.043	0.306
	High BP	miR-543	Yes	Primary	-0.13 (-0.25, 0)	0.049	0.320
	Moderate High BP	hsa-miR-1972	No	Primary	0.27 (0.03, 0.51)	0.029	0.242
	Moderate High BP	hsa-miR-23a-3p	No	Primary	-0.31 (-0.59, -0.02)	0.041	0.299
	Moderate High BP	hsa-miR-210-3p	No	Primary	-0.21 (-0.33, -0.08)	0.002	0.137
	Moderate High BP	hsa-miR-150-5p	No	Primary	0.15 (0.05, 0.25)	0.003	0.137
	Moderate Low	hsa-miR-199b-5p	No	Primary	-0.20 (-0.32, -0.07)	0.003	0.137
	Moderate Low	hsa-miR-331-3p	No	Primary	-0.18 (-0.3, -0.06)	0.003	0.137
	Moderate Low	hsa-miR-1272	No	Primary	-0.21 (-0.36, -0.07)	0.005	0.151

	Moderate Low	hsa-miR-26a-5p	No	Primary	-0.12 (-0.21, -0.03)	0.007	0.151
	Moderate Low	hsa-let-7b-5p	No	Primary	0.08 (0.02, 0.14)	0.009	0.163
	Moderate Low	hsa-miR-188-5p	No	Primary	-0.16 (-0.27, -0.04)	0.009	0.163
	Moderate Low	hsa-miR-29b-3p	No	Primary	0.09 (0.02, 0.17)	0.013	0.179
	Moderate Low	hsa-miR-4516	No	Primary	0.14 (0.03, 0.25)	0.016	0.179
	Moderate Low	hsa-miR-302b-3p	No	Primary	-0.11 (-0.21, -0.02)	0.019	0.192
	Moderate Low	hsa-miR-320e	No	Primary	0.16 (0.03, 0.29)	0.019	0.192
	Moderate Low	hsa-miR-107	No	Primary	-0.08 (-0.15, -0.01)	0.021	0.203
	Moderate Low	hsa-miR-221-3p	No	Primary	0.09 (0.01, 0.18)	0.038	0.299
	Moderate Low	hsa-miR-608	No	Primary	-0.10 (-0.2, -0.01)	0.040	0.299
	Moderate Low	hsa-miR-937-3p	No	Primary	-0.11 (-0.22, 0)	0.045	0.314
	Moderate Low	hsa-miR-4454+hsa-miR-7975	No	Primary	0.10 (0, 0.19)	0.047	0.319
	**Moderate Low**	**hsa-miR-526a+hsa-miR-518c-5p+hsa-miR-518d-5p**	**Yes**	**Secondary**	**-0.46 (-0.77, -0.15)**	**0.004**	**0.043**
	Moderate Low	hsa-miR-1185-5p	Yes	Secondary	-0.33 (-0.63, -0.03)	0.032	0.184
	**Moderate Low**	**hsa-miR-300**	**Yes**	**Secondary**	**-0.18 (-0.29, -0.07)**	**0.001**	**0.041**
	**Moderate Low**	**hsa-miR-519c-3p**	**Yes**	**Secondary**	**-0.14 (-0.23, -0.05)**	**0.002**	**0.041**
	**Moderate Low**	**hsa-miR-526a+hsa-miR-518c-5p+hsa-miR-518d-5p**	**Yes**	**Secondary**	**-0.17 (-0.27, -0.06)**	**0.003**	**0.041**
	**Moderate Low**	**hsa-miR-770-5p**	**Yes**	**Secondary**	**-0.15 (-0.24, -0.05)**	**0.003**	**0.041**
	Moderate Low	hsa-miR-432-5p	Yes	Secondary	-0.15 (-0.26, -0.04)	0.007	0.053
	Moderate Low	hsa-miR-370-3p	Yes	Secondary	-0.15 (-0.26, -0.04)	0.007	0.053
	Moderate Low	hsa-miR-517a-3p	Yes	Secondary	-0.14 (-0.24, -0.03)	0.010	0.062
	Moderate Low	hsa-miR-485-5p	Yes	Secondary	-0.09 (-0.18, 0)	0.047	0.245

Table S6: Sensitivity Analyses

Bolded rows highlight miRNAs with PFDR < 0.05. This table shows the results of sensitivity analyses which either exclude participants based on covariate information (orange text) or additionally adjust for covariates in statistical models (red text). Reference effect estimates and p-values are indicated by green text.

Predictor	Outcome	Primary or Secondary Outcome	Effect Estimate (95% CI)	P-value	P(FDR)	Effect Estimate (95% CI)	P-value	P(FDR)	% Change of the Effect Estimate	Effect Estimate (95% CI)	P-value	P(FDR)	% Change of the Effect Estimate
			Primary Analysis (n=372)			Excluding participants with diabetes (n=321)				Excluding participants that didn't report using prenatal vitamins (n=363)			
Hypertensive Subtypes													
Chronic Hypertension	miR-608	Primary	-0.31 (-0.59, -0.03)	0.03	0.92	-0.39 (-0.70, -0.08)	0.01	0.80	-26%	-0.40 (-0.70, -0.11)	0.01	0.68	-29%
Preeclampsia/Eclampsia	miR-1257	Primary	-0.17 (-0.29, -0.05)	0.01	0.92	-0.13 (-0.37, 0.14)	0.07	0.80	24%	-0.15 (-0.37, 0.13)	0.02	0.73	12%
Preeclampsia/Eclampsia	miR-1262	Primary	-0.19 (-0.34, -0.04)	0.01	0.92	-0.22 (-0.41, 0.23)	0.02	0.80	-16%	-0.20 (-0.41, 0.20)	0.01	0.68	-5%
Preeclampsia/Eclampsia	miR-584-5p	Primary	-0.15 (-0.29, -0.02)	0.03	0.92	-0.15 (-0.39, 0.19)	0.08	0.80	0%	-0.16 (-0.37, 0.19)	0.03	0.73	-7%
Preeclampsia/Eclampsia	miR-585-3p	Primary	-0.18 (-0.34, -0.02)	0.03	0.92	-0.20 (-0.42, 0.26)	0.04	0.80	-11%	-0.16 (-0.41, 0.25)	0.06	0.73	11%
Preeclampsia/Eclampsia	miR-122-5p	Primary	-0.19 (-0.38, -0.01)	0.05	0.92	-0.15 (-0.29, 0.49)	0.18	0.80	21%	-0.23 (-0.28, 0.48)	0.02	0.73	-21%
Preeclampsia/Eclampsia	miR-1972	Primary	-0.13 (-0.26, 0)	0.05	0.92	-0.13 (-0.42, 0.11)	0.08	0.80	0%	-0.16 (-0.42, 0.10)	0.02	0.73	-23%
Preeclampsia/Eclampsia	miR-656-3p	Primary	-0.13 (-0.26, -0.01)	0.05	0.92	-0.14 (-0.36, 0.17)	0.07	0.80	-8%	-0.14 (-0.36, 0.16)	0.04	0.73	-8%
Chronic Hypertension	**miR-1185-2-3p**	**Secondary**	**0.50 (0.24, 0.77)**	**0.00**	**0.02**	0.45 (0.17, 0.73)	0.00	0.00	-10%	0.47 (0.19, 0.74)	0.00	0.00	-6%
Chronic Hypertension	miR-520h	Secondary	-0.34 (-0.66, -0.03)	0.03	0.71	-0.32 (-0.65, 0.02)	0.06	0.78	6%	-0.33 (-0.66, -0.00)	0.05	0.66	3%
Chronic Hypertension	miR-382-5p	Secondary	0.33 (0.01, 0.65)	0.04	0.71	0.39 (0.05, 0.72)	0.03	0.78	18%	0.40 (0.07, 0.74)	0.02	0.66	21%
Gestational Hypertension	miR-370-3p	Secondary	-0.23 (-0.45, -0.01)	0.04	0.71	-0.28 (-0.39, 0.28)	0.03	0.78	-22%	-0.23 (-0.38, 0.28)	0.05	0.66	0%
BP Trajectories													
High BP	miR-331-3p	Primary	-0.31 (-0.51, -0.11)	0.00	0.24	-0.30 (-0.09, 0.34)	0.00	0.00	3%	-0.31 (-0.08, 0.33)	0.00	0.00	0%
High BP	miR-656-3p	Primary	-0.22 (-0.36, -0.07)	0.00	0.24	-0.20 (-0.03, 0.29)	0.01	0.34	9%	-0.23 (-0.02, 0.28)	0.00	0.00	-5%
High BP	miR-1272	Primary	-0.31 (-0.56, -0.06)	0.01	0.24	-0.31 (-0.12, 0.45)	0.02	0.34	0%	-0.33 (-0.08, 0.44)	0.01	0.18	-6%
High BP	miR-199b-5p	Primary	-0.28 (-0.5, -0.07)	0.01	0.24	-0.25 (-0.12, 0.36)	0.03	0.34	11%	-0.30 (-0.12, 0.33)	0.01	0.18	-7%
High BP	miR-210-3p	Primary	-0.28 (-0.5, -0.06)	0.01	0.24	-0.29 (-0.12, 0.37)	0.02	0.34	-4%	-0.29 (-0.11, 0.36)	0.01	0.18	-4%
High BP	miR-212-3p	Primary	-0.23 (-0.39, -0.06)	0.01	0.24	-0.22 (-0.16, 0.21)	0.01	0.34	4%	-0.24 (-0.13, 0.22)	0.01	0.18	-4%
High BP	miR-221-3p	Primary	0.18 (0.04, 0.33)	0.01	0.24	0.16 (-0.23, 0.10)	0.05	0.47	-11%	0.19 (-0.21, 0.10)	0.01	0.18	6%
High BP	miR-302b-3p	Primary	-0.21 (-0.37, -0.06)	0.01	0.24	-0.22 (-0.15, 0.20)	0.01	0.34	-5%	-0.22 (-0.11, 0.22)	0.01	0.18	-5%
High BP	miR-607	Primary	-0.19 (-0.34, -0.04)	0.01	0.24	-0.18 (-0.11, 0.23)	0.03	0.34	5%	-0.20 (-0.10, 0.21)	0.01	0.18	-5%
High BP	miR-608	Primary	-0.20 (-0.36, -0.04)	0.01	0.24	-0.19 (-0.15, 0.22)	0.03	0.34	5%	-0.22 (-0.12, 0.22)	0.01	0.18	-10%
High BP	miR-1262	Primary	-0.18 (-0.35, -0.02)	0.03	0.38	-0.20 (-0.20, 0.17)	0.03	0.34	-11%	-0.20 (-0.16, 0.19)	0.02	0.23	-11%
High BP	miR-188-5p	Primary	-0.22 (-0.41, -0.03)	0.03	0.38	-0.23 (-0.10, 0.33)	0.03	0.34	-5%	-0.23 (-0.08, 0.32)	0.02	0.23	-5%
High BP	miR-4454+miR-7975	Primary	0.18 (0.02, 0.34)	0.03	0.38	0.19 (-0.25, 0.10)	0.03	0.34	6%	0.19 (-0.24, 0.10)	0.02	0.23	6%
High BP	miR-1236-3p	Primary	-0.16 (-0.31, -0.01)	0.04	0.42	-0.18 (-0.18, 0.16)	0.03	0.34	-13%	-0.17 (-0.14, 0.17)	0.03	0.30	-6%
High BP	miR-1290	Primary	-0.14 (-0.27, -0.01)	0.04	0.41	-0.16 (-0.13, 0.17)	0.02	0.34	-14%	-0.15 (-0.09, 0.19)	0.02	0.23	-7%
High BP	miR-148a-3p	Primary	0.13 (0.00, 0.26)	0.04	0.42	0.13 (-0.18, 0.10)	0.06	0.47	0%	0.13 (-0.18, 0.09)	0.04	0.30	0%
High BP	miR-585-3p	Primary	-0.19 (-0.36, -0.01)	0.04	0.41	-0.20 (-0.22, 0.18)	0.04	0.40	-5%	-0.21 (-0.18, 0.19)	0.02	0.23	-11%
High BP	miR-107	Primary	-0.11 (-0.22, 0)	0.05	0.43	-0.07 (-0.15, 0.10)	0.22	0.62	36%	-0.11 (-0.16, 0.08)	0.06	0.33	0%
Moderate BP	miR-130a-3p	Primary	-0.29 (-0.5, -0.07)	0.01	0.24	-0.30 (-0.54, -0.07)	0.01	0.34	-3%	-0.28 (-0.49, -0.06)	0.02	0.23	3%
Moderate BP	miR-1257	Primary	0.15 (0.01, 0.29)	0.04	0.42	0.14 (-0.01, 0.29)	0.07	0.47	-7%	0.14 (-0.00, 0.28)	0.05	0.33	-7%
Moderate BP	miR-485-3p	Secondary	-0.23 (-0.41, -0.05)	0.01	0.29	-0.22 (-0.41, -0.03)	0.02	0.35	4%	-0.23 (-0.40, -0.05)	0.01	0.26	0%
Moderate BP	miR-526a+miR-518c-5p+miR-518d-5p	Secondary	-0.26 (-0.45, -0.07)	0.01	0.29	-0.28 (-0.48, -0.08)	0.01	0.26	-8%	-0.25 (-0.44, -0.06)	0.01	0.26	4%
Moderate BP	miR-323b-3p	Secondary	-0.18 (-0.34, -0.03)	0.02	0.30	-0.19 (-0.36, -0.02)	0.03	0.39	-6%	-0.18 (-0.34, -0.03)	0.02	0.26	0%
Moderate BP	miR-411-5p	Secondary	-0.23 (-0.42, -0.04)	0.02	0.29	-0.26 (-0.45, -0.06)	0.01	0.26	-13%	-0.23 (-0.41, -0.04)	0.02	0.26	0%
		Average Percent Change:							-1%				-3%

Variable Description:

All analyses were adjusted for maternal age, perceived stress during pregnancy, BMI, recruitment site, and timing of enrollment.

Diabetes status during pregnancy was determined using information from the maternal EMRs and self-reported questionnaires, as described previously in Howe, C. G. et al. Extracellular vesicle microRNA in early versus late pregnancy with birth outcomes in the MADRES study. Epigenetics 17, 269 (2022).

Prenatal vitamin use was collected by questionnaire during pregnancy

Effect Estimate (95% CI)	P-value	P(FDR)	% Change of the Effect Estimate	Effect Estimate (95% CI)	P-value	P(FDR)	% Change of the Effect Estimate	Effect Estimate (95% CI)	P-value	P(FDR)	% Change of the Effect Estimate	Effect Estimate (95% CI)	P-value	P(FDR)	% Change of the Effect Estimate	Effect Estimate (95% CI)	P-value	P(FDR)	% Change of the Effect Estimate
Excluding non-fasting participants (n=366)				Excluding participants with chronic hypertension (n=362)				Excluding participants that reported smoking during pregnancy (n=365)				Excluding participants with any hypertension (n=300)				Excluding participants using blood pressure medication during pregnancy (n=341)			
-0.29 (-0.59, 0.01)	0.06	0.74	6%	NA	NA	NA	NA	-0.35 (-0.64, -0.07)	0.01	0.68	-13%	NA	NA	NA	NA	-0.32 (-0.60, -0.03)	0.03	0.52	-3%
-0.17 (-0.31, 0.19)	0.01	0.74	0%	-0.17 (-0.29, -0.05)	0.01	0.63	0%	-0.17 (-0.37, 0.11)	0.01	0.68	0%	NA	NA	NA	NA	-0.18 (-0.34, 0.12)	0.01	0.52	-6%
-0.21 (-0.28, 0.33)	0.01	0.74	-11%	-0.20 (-0.35, -0.04)	0.01	0.63	-5%	-0.19 (-0.35, 0.24)	0.01	0.68	0%	NA	NA	NA	NA	-0.23 (-0.34, 0.25)	0.01	0.52	-21%
-0.17 (-0.25, 0.30)	0.02	0.74	-13%	-0.16 (-0.30, -0.02)	0.02	0.63	-7%	-0.16 (-0.32, 0.21)	0.02	0.74	-7%	NA	NA	NA	NA	-0.17 (-0.31, 0.21)	0.03	0.52	-13%
-0.18 (-0.26, 0.38)	0.03	0.74	0%	-0.19 (-0.35, -0.03)	0.02	0.63	-6%	-0.19 (-0.34, 0.29)	0.02	0.74	-6%	NA	NA	NA	NA	-0.25 (-0.32, 0.30)	0.00	0.00	-39%
-0.22 (-0.25, 0.50)	0.03	0.74	-16%	-0.20 (-0.38, -0.01)	0.05	0.63	-5%	-0.19 (-0.27, 0.43)	0.05	0.74	0%	NA	NA	NA	NA	-0.12 (-0.27, 0.44)	0.27	0.76	37%
-0.16 (-0.34, 0.18)	0.02	0.74	-23%	-0.14 (-0.27, -0.01)	0.04	0.63	-8%	-0.14 (-0.37, 0.12)	0.04	0.74	-8%	NA	NA	NA	NA	-0.16 (-0.37, 0.13)	0.02	0.52	-23%
-0.13 (-0.28, 0.23)	0.05	0.74	0%	-0.15 (-0.27, -0.02)	0.03	0.63	-15%	-0.15 (-0.34, 0.15)	0.03	0.74	-15%	NA	NA	NA	NA	-0.17 (-0.31, 0.18)	0.02	0.52	-31%
0.47 (0.19, 0.74)	0.00	0.00	-6%	NA	NA	NA	NA	0.50 (0.24, 0.77)	0.00	0.00	0%	NA	NA	NA	NA	0.49 (0.22, 0.76)	0.00	0.00	-2%
-0.33 (-0.66, 0.00)	0.05	0.70	3%	NA	NA	NA	NA	-0.34 (-0.65, -0.02)	0.04	0.70	0%	NA	NA	NA	NA	-0.36 (-0.68, -0.04)	0.03	0.62	-6%
0.29 (-0.05, 0.62)	0.09	0.70	-12%	NA	NA	NA	NA	0.34 (0.02, 0.65)	0.04	0.70	3%	NA	NA	NA	NA	0.34 (0.02, 0.66)	0.04	0.62	3%
-0.23 (-0.38, 0.29)	0.04	0.70	0%	-0.22 (-0.31, 0.02)	0.05	0.60	4%	-0.23 (-0.31, 0.33)	0.04	0.70	0%	NA	NA	NA	NA	-0.34 (-0.30, 0.33)	0.01	0.39	-48%
-0.32 (-0.08, 0.33)	0.00	0.00	-3%	-0.28 (-0.06, 0.37)	0.01	0.36	10%	-0.31 (-0.09, 0.33)	0.00	0.00	0%	-0.23 (-0.13, 0.42)	0.26	0.99	26%	-0.30 (-0.12, 0.32)	0.01	0.45	3%
-0.22 (-0.02, 0.29)	0.00	0.00	0%	-0.22 (-0.02, 0.29)	0.00	0.00	0%	-0.22 (-0.03, 0.27)	0.00	0.00	0%	-0.03 (-0.08, 0.29)	0.84	0.99	86%	-0.21 (-0.05, 0.25)	0.01	0.45	5%
-0.31 (-0.07, 0.44)	0.02	0.26	0%	-0.30 (-0.06, 0.48)	0.02	0.36	3%	-0.32 (-0.08, 0.44)	0.01	0.18	-3%	-0.17 (-0.06, 0.64)	0.51	0.99	45%	-0.30 (-0.13, 0.43)	0.03	0.49	3%
-0.27 (-0.11, 0.34)	0.02	0.26	4%	-0.31 (-0.12, 0.36)	0.01	0.36	-11%	-0.29 (-0.12, 0.34)	0.01	0.18	-4%	-0.16 (-0.17, 0.45)	0.48	0.99	43%	-0.24 (-0.16, 0.32)	0.04	0.53	14%
-0.28 (-0.10, 0.36)	0.01	0.20	0%	-0.26 (-0.09, 0.40)	0.03	0.42	7%	-0.28 (-0.11, 0.36)	0.01	0.18	0%	-0.15 (-0.11, 0.50)	0.49	0.99	46%	-0.26 (-0.17, 0.32)	0.03	0.49	7%
-0.22 (-0.12, 0.22)	0.01	0.20	4%	-0.21 (-0.12, 0.24)	0.02	0.36	9%	-0.23 (-0.13, 0.22)	0.01	0.18	0%	-0.08 (-0.18, 0.27)	0.61	0.99	65%	-0.22 (-0.15, 0.21)	0.02	0.49	4%
0.19 (-0.21, 0.10)	0.01	0.20	6%	0.18 (-0.24, 0.08)	0.02	0.36	0%	0.18 (-0.21, 0.10)	0.02	0.30	0%	0.16 (-0.32, 0.10)	0.28	0.99	-11%	0.18 (-0.21, 0.11)	0.02	0.49	0%
-0.22 (-0.11, 0.22)	0.01	0.20	-5%	-0.21 (-0.12, 0.23)	0.01	0.36	0%	-0.22 (-0.11, 0.22)	0.01	0.18	-5%	-0.19 (-0.17, 0.27)	0.23	0.99	10%	-0.22 (-0.16, 0.19)	0.01	0.45	-5%
-0.19 (-0.09, 0.22)	0.01	0.20	0%	-0.18 (-0.09, 0.23)	0.03	0.42	5%	-0.19 (-0.10, 0.21)	0.01	0.18	0%	-0.18 (-0.11, 0.30)	0.23	0.99	5%	-0.21 (-0.13, 0.20)	0.01	0.45	-11%
-0.21 (-0.12, 0.22)	0.01	0.20	-5%	-0.20 (-0.12, 0.24)	0.02	0.36	0%	-0.21 (-0.12, 0.22)	0.01	0.18	-5%	-0.04 (-0.12, 0.33)	0.80	0.99	80%	-0.20 (-0.16, 0.20)	0.03	0.49	0%
-0.20 (-0.16, 0.19)	0.02	0.26	-11%	-0.17 (-0.18, 0.18)	0.05	0.43	6%	-0.18 (-0.17, 0.19)	0.03	0.34	0%	0.09 (-0.36, 0.09)	0.57	0.99	150%	-0.18 (-0.17, 0.20)	0.05	0.53	0%
-0.23 (-0.07, 0.33)	0.02	0.26	-5%	-0.19 (-0.07, 0.35)	0.07	0.48	14%	-0.22 (-0.07, 0.33)	0.02	0.30	0%	-0.17 (-0.16, 0.38)	0.40	0.99	23%	-0.19 (-0.10, 0.33)	0.07	0.60	14%
0.18 (-0.24, 0.09)	0.03	0.33	0%	0.21 (-0.25, 0.10)	0.02	0.36	17%	0.18 (-0.23, 0.10)	0.03	0.34	0%	0.19 (-0.45, -0.01)	0.23	0.99	6%	0.17 (-0.26, 0.09)	0.06	0.54	-6%
-0.17 (-0.14, 0.18)	0.03	0.33	-6%	-0.13 (-0.16, 0.18)	0.10	0.53	19%	-0.16 (-0.14, 0.18)	0.04	0.40	0%	0.02 (-0.21, 0.21)	0.89	0.99	113%	-0.15 (-0.15, 0.18)	0.06	0.54	6%
-0.16 (-0.09, 0.19)	0.02	0.26	-14%	-0.15 (-0.10, 0.20)	0.03	0.42	-7%	-0.14 (-0.10, 0.18)	0.03	0.34	0%	0.00 (-0.18, 0.19)	0.99	0.99	100%	-0.14 (-0.15, 0.14)	0.05	0.53	0%
0.14 (-0.18, 0.09)	0.04	0.33	8%	0.14 (-0.19, 0.09)	0.04	0.43	8%	0.13 (-0.18, 0.08)	0.05	0.43	0%	0.14 (-0.29, 0.05)	0.26	0.99	8%	0.12 (-0.18, 0.10)	0.10	0.64	-8%
-0.19 (-0.18, 0.19)	0.04	0.33	0%	-0.17 (-0.20, 0.18)	0.07	0.48	11%	-0.19 (-0.19, 0.19)	0.03	0.34	0%	-0.02 (-0.25, 0.21)	0.90	0.99	89%	-0.18 (-0.23, 0.16)	0.06	0.54	5%
-0.09 (-0.16, 0.08)	0.10	0.42	18%	-0.12 (-0.18, 0.06)	0.05	0.43	-9%	-0.11 (-0.16, 0.07)	0.04	0.40	0%	-0.11 (-0.16, 0.14)	0.34	0.99	0%	-0.14 (-0.15, 0.09)	0.02	0.49	-27%
-0.29 (-0.51, -0.07)	0.01	0.20	0%	-0.27 (-0.49, -0.04)	0.02	0.36	7%	-0.28 (-0.49, -0.06)	0.01	0.18	3%	-0.26 (-0.54, 0.02)	0.07	0.99	10%	-0.24 (-0.47, -0.01)	0.04	0.53	17%
0.15 (0.01, 0.29)	0.04	0.33	0%	0.15 (-0.00, 0.29)	0.05	0.43	0%	0.14 (-0.00, 0.28)	0.06	0.43	-7%	0.05 (-0.14, 0.23)	0.62	0.99	-67%	0.12 (-0.03, 0.26)	0.12	0.65	-20%
-0.23 (-0.41, -0.05)	0.01	0.17	0%	-0.21 (-0.40, -0.02)	0.03	0.52	9%	-0.23 (-0.41, -0.05)	0.01	0.26	0%	-0.24 (-0.48, -0.01)	0.05	0.80	-4%	-0.21 (-0.40, -0.02)	0.03	0.52	9%
-0.26 (-0.45, -0.07)	0.01	0.17	0%	-0.26 (-0.46, -0.06)	0.01	0.52	0%	-0.26 (-0.45, -0.07)	0.01	0.26	0%	-0.24 (-0.48, 0.01)	0.06	0.80	8%	-0.23 (-0.43, -0.03)	0.02	0.52	12%
-0.18 (-0.33, -0.02)	0.03	0.39	0%	-0.17 (-0.33, -0.00)	0.05	0.52	6%	-0.18 (-0.33, -0.02)	0.03	0.39	0%	-0.14 (-0.35, 0.08)	0.22	0.80	22%	-0.17 (-0.33, -0.00)	0.05	0.62	6%
-0.23 (-0.42, -0.05)	0.01	0.17	0%	-0.20 (-0.40, -0.01)	0.04	0.52	13%	-0.23 (-0.42, -0.04)	0.02	0.35	0%	-0.21 (-0.46, 0.04)	0.10	0.80	9%	-0.22 (-0.42, -0.03)	0.03	0.52	4%
			-2%				5%				-2%				36%				1%
At the time of sample collection, participants reported if they were fasting				Participants were classified as having HDP based on a physician diagnosis in the EMRs or BP measures abstracted from the EMRs, as described in the manuscript				Smoking status was collected by questionnaire during pregnancy				Participants were classified as having HDP based on a physician diagnosis in the EMRs or BP measures abstracted from the EMRs, as described in the manuscript				Medication use was collected by questionnaire during pregnancy. A participant was categorized as using a medication to control blood pressure during pregnancy if at any time they indicated they were using Aspirin, Methyldopa (Aldomet), Labetalol (Normodyne, Trandate), Nifedipine (Procardia, Adalat), or Hydralazine.			

Effect Estimate (95% CI)	P-value	P(FDR)	% Change of the Effect Estimate	Effect Estimate (95% CI)	P-value	P(FDR)	% Change of the Effect Estimate
Additionally adjusting for gestational weight gain (n=372)				Additionally adjusting for gestational age in weeks at blood collection (n=372)			
-0.35 (-0.63,-0.06)	0.02	0.78	-13%	-0.35 (-0.63,-0.06)	0.02	0.81	-13%
-0.17 (-0.36,0.12)	0.01	0.78	0%	-0.16 (-0.36,0.12)	0.01	0.81	6%
-0.18 (-0.34,0.25)	0.02	0.78	5%	-0.19 (-0.34,0.25)	0.02	0.81	0%
-0.16 (-0.31,0.22)	0.03	0.78	-7%	-0.15 (-0.31,0.22)	0.03	0.81	0%
-0.19 (-0.34,0.29)	0.03	0.78	-6%	-0.18 (-0.33,0.29)	0.03	0.81	0%
-0.20 (-0.27,0.44)	0.05	0.78	-5%	-0.18 (-0.27,0.44)	0.06	0.81	5%
-0.13 (-0.37,0.13)	0.05	0.78	0%	-0.14 (-0.37,0.12)	0.04	0.81	-8%
-0.14 (-0.33,0.16)	0.05	0.78	-8%	-0.14 (-0.33,0.16)	0.04	0.81	-8%
0.49 (0.23,0.76)	0.00	0.00	-2%	0.50 (0.24,0.77)	0.00	0.00	0%
-0.34 (-0.66,-0.02)	0.04	0.66	0%	-0.34 (-0.66,-0.03)	0.03	0.71	0%
0.33 (0.01,0.65)	0.04	0.66	0%	0.33 (0.02,0.65)	0.04	0.71	0%
-0.23 (-0.32,0.32)	0.04	0.66	0%	-0.24 (-0.31,0.33)	0.04	0.71	-4%
-0.32 (-0.10,0.31)	0.00	0.00	-3%	-0.31 (-0.08,0.33)	0.00	0.00	0%
-0.21 (-0.01,0.29)	0.00	0.00	5%	-0.22 (-0.01,0.28)	0.00	0.00	0%
-0.31 (-0.09,0.44)	0.01	0.20	0%	-0.31 (-0.08,0.44)	0.01	0.16	0%
-0.29 (-0.13,0.33)	0.01	0.20	-4%	-0.29 (-0.11,0.33)	0.01	0.16	-4%
-0.28 (-0.12,0.36)	0.01	0.20	0%	-0.28 (-0.11,0.36)	0.01	0.16	0%
-0.24 (-0.14,0.21)	0.01	0.20	-4%	-0.23 (-0.12,0.22)	0.01	0.16	0%
0.17 (-0.22,0.09)	0.02	0.30	-6%	0.18 (-0.21,0.10)	0.01	0.16	0%
-0.20 (-0.10,0.23)	0.01	0.20	5%	-0.22 (-0.11,0.22)	0.01	0.16	-5%
-0.19 (-0.10,0.21)	0.01	0.20	0%	-0.19 (-0.10,0.21)	0.01	0.16	0%
-0.19 (-0.11,0.24)	0.02	0.30	5%	-0.21 (-0.12,0.22)	0.01	0.16	-5%
-0.17 (-0.15,0.21)	0.04	0.38	6%	-0.19 (-0.16,0.19)	0.03	0.34	-6%
-0.21 (-0.07,0.34)	0.03	0.38	5%	-0.22 (-0.08,0.33)	0.03	0.34	0%
0.20 (-0.22,0.12)	0.02	0.30	11%	0.18 (-0.24,0.09)	0.03	0.34	0%
-0.15 (-0.14,0.18)	0.05	0.39	6%	-0.16 (-0.14,0.18)	0.04	0.36	0%
-0.14 (-0.09,0.19)	0.04	0.38	0%	-0.14 (-0.09,0.18)	0.03	0.34	0%
0.14 (-0.17,0.10)	0.03	0.38	8%	0.13 (-0.18,0.09)	0.04	0.36	0%
-0.19 (-0.18,0.20)	0.04	0.38	0%	-0.19 (-0.17,0.20)	0.03	0.34	0%
-0.11 (-0.16,0.08)	0.06	0.40	0%	-0.11 (-0.16,0.07)	0.04	0.36	0%
-0.30 (-0.52,-0.09)	0.01	0.20	-3%	-0.29 (-0.50,-0.07)	0.01	0.16	0%
0.15 (0.01,0.30)	0.04	0.38	0%	0.15 (0.01,0.29)	0.04	0.36	0%
-0.23 (-0.41,-0.04)	0.02	0.39	0%	-0.23 (-0.41,-0.05)	0.01	0.26	**0%**
-0.25 (-0.44,-0.05)	0.01	0.39	4%	-0.26 (-0.45,-0.07)	0.01	0.26	0%
-0.18 (-0.34,-0.02)	0.03	0.39	0%	-0.18 (-0.34,-0.03)	0.02	0.26	0%
-0.21 (-0.40,-0.02)	0.03	0.39	9%	-0.23 (-0.42,-0.04)	0.02	0.26	0%
			0%				-1%

Total gestational weight gain was calculated by taking the difference between the last recorded weight (up to two weeks after delivery) and the pre-pregnancy BMI, with measures sourced from questionnaires, collected by study staff, and abstracted from EMRs.

Gestational age in weeks at sample collection was calculated based on the date of blood collection, the gestational age at birth, and the date of birth.

Effect Estimate (95% CI)	P-value	P(FDR)	Effect Estimate (95% CI)	P-value	P(FDR)	% Change of the Effect Estimate
Primary model restricting to participants with measures of physical activity (n=370)			Additionally adjusting for physical activity during pregnancy (n=370)			
-0.35 (-0.63, -0.07)	0.02	0.76	-0.35 (-0.64,-0.07)	0.01	0.68	0%
-0.17 (-0.36, 0.12)	0.01	0.76	-0.16 (-0.36,0.11)	0.01	0.68	6%
-0.19 (-0.34, 0.24)	0.01	0.76	-0.19 (-0.34,0.24)	0.01	0.68	0%
-0.16 (-0.31, 0.22)	0.02	0.76	-0.16 (-0.31,0.22)	0.02	0.85	0%
-0.18 (-0.33, 0.29)	0.03	0.76	-0.18 (-0.33,0.29)	0.03	0.85	0%
-0.19 (-0.27, 0.45)	0.05	0.76	-0.19 (-0.27,0.45)	0.06	0.85	0%
-0.14 (-0.37, 0.12)	0.04	0.76	-0.13 (-0.37,0.12)	0.05	0.85	7%
-0.14 (-0.33, 0.16)	0.03	0.76	-0.14 (-0.33,0.16)	0.04	0.85	0%
0.50 (0.24, 0.77)	0.00	0.00	0.50 (0.24,0.77)	0.00	0.00	0%
-0.34 (-0.66, -0.03)	0.03	0.71	-0.34 (-0.66,-0.03)	0.03	0.70	0%
0.33 (0.01, 0.65)	0.04	0.71	0.33 (0.01,0.65)	0.04	0.70	0%
-0.23 (-0.31, 0.33)	0.04	0.71	-0.23 (-0.31,0.33)	0.05	0.70	0%
-0.31 (-0.09, 0.33)	0.00	0.00	-0.30 (-0.07,0.33)	0.00	0.00	3%
-0.22 (-0.02, 0.28)	0.00	0.00	-0.21 (-0.01,0.28)	0.00	0.00	5%
-0.31 (-0.09, 0.43)	0.01	0.16	-0.29 (-0.08,0.44)	0.02	0.33	6%
-0.29 (-0.12, 0.33)	0.01	0.16	-0.28 (-0.11,0.33)	0.01	0.23	3%
-0.29 (-0.12, 0.35)	0.01	0.16	-0.27 (-0.09,0.36)	0.02	0.33	7%
-0.23 (-0.13, 0.21)	0.01	0.16	-0.22 (-0.12,0.23)	0.01	0.23	4%
0.18 (-0.20, 0.10)	0.01	0.16	0.17 (-0.21,0.08)	0.02	0.33	-6%
-0.22 (-0.11, 0.21)	0.01	0.16	-0.21 (-0.11,0.22)	0.01	0.23	5%
-0.19 (-0.10, 0.21)	0.01	0.16	-0.18 (-0.09,0.22)	0.01	0.23	5%
-0.21 (-0.12, 0.22)	0.01	0.16	-0.20 (-0.12,0.22)	0.01	0.23	5%
-0.19 (-0.16, 0.19)	0.03	0.34	-0.18 (-0.16,0.19)	0.03	0.39	5%
-0.22 (-0.08, 0.32)	0.02	0.30	-0.21 (-0.07,0.33)	0.03	0.39	5%
0.18 (-0.24, 0.09)	0.03	0.34	0.18 (-0.24,0.09)	0.03	0.39	0%
-0.16 (-0.14, 0.18)	0.04	0.40	-0.16 (-0.14,0.18)	0.05	0.41	0%
-0.14 (-0.09, 0.18)	0.03	0.34	-0.14 (-0.09,0.19)	0.04	0.40	0%
0.13 (-0.17, 0.09)	0.04	0.40	0.12 (-0.18,0.08)	0.05	0.41	-8%
-0.19 (-0.18, 0.19)	0.03	0.34	-0.18 (-0.17,0.20)	0.04	0.40	5%
-0.11 (-0.16, 0.08)	0.05	0.40	-0.11 (-0.16,0.08)	0.05	0.41	0%
-0.28 (-0.49, -0.06)	0.01	0.16	-0.28 (-0.50,-0.06)	0.01	0.23	0%
0.14 (0.00, 0.29)	0.05	0.40	0.15 (0.01,0.29)	0.04	0.40	7%
-0.23 (-0.41, -0.05)	0.01	0.26	-0.23 (-0.41,-0.05)	0.01	0.26	0%
-0.26 (-0.45, -0.07)	0.01	0.26	-0.26 (-0.45,-0.07)	0.01	0.26	0%
-0.18 (-0.34, -0.03)	0.02	0.26	-0.19 (-0.34,-0.03)	0.02	0.26	-6%
-0.23 (-0.41, -0.04)	0.02	0.26	-0.23 (-0.41,-0.04)	0.02	0.26	0%
						2%

Physical activity during pregnancy was measured using the validated pregnancy physical activity questionnaire (PPAQ). This questionnaire was administered during the participant's first study visit and asked participants to recall the frequency and duration of 32 physical activities that range from sedentary to vigorous. A total score representing the metabolic equivalent (MET) hours per week was calculated from these reported physical activities for each participant.

Figure S1: Participant Selection

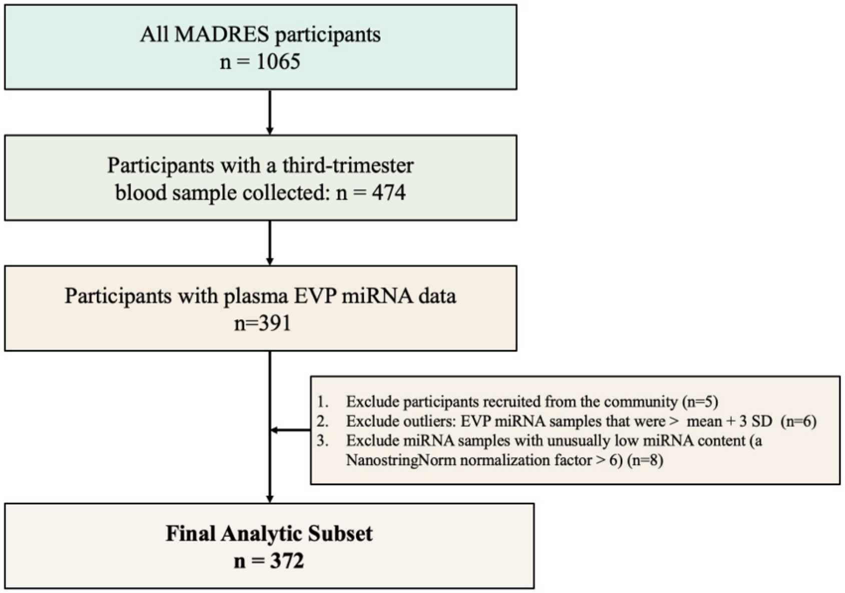

This flowchart illustrates the process of participant selection for the analytic subset from participants enrolled in the MADRES cohort as of July 16, 2023. Participants who provided a third-trimester blood sample and had plasma EVP miRNA measures available for this time point were included in the analytic subset. EVP miRNA counts were normalized to sample-specific positive controls included on the nCounter platform using the NanoStringNorm package (v1.2.1.1). Samples flagged as having abnormally high or low levels of miRNAs were excluded from subsequent analyses. 372 participants were included in the final analytic subset.

Figure S2: Diastolic Blood Pressure Trajectories

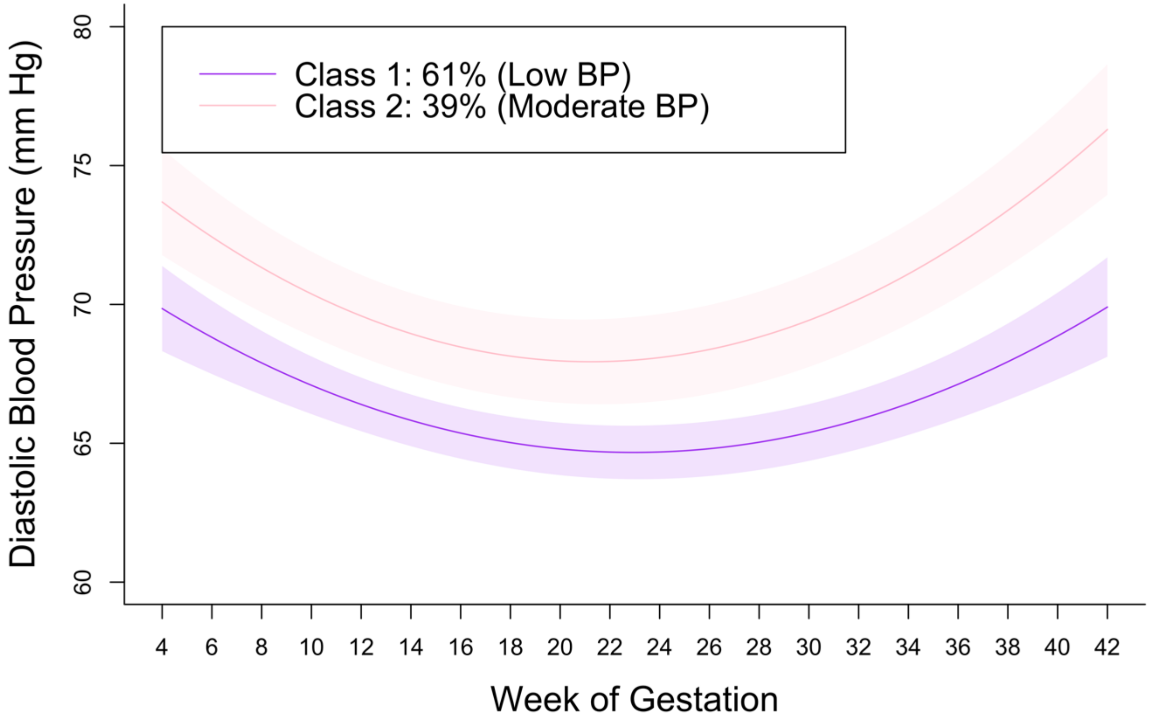

When latent class trajectory modeling was conducted with the same parameters as the k=3 SBP trajectories, we identified two diastolic blood pressure (DBP) trajectories (the BIC for a k of 2 was 71957.51). These two trajectories are plotted with their median and 95% confidence interval bands: (1) Low BP and (2) Moderate BP. Both trajectories parallel each other throughout gestation, starting with a median DBP between 70-75 mm Hg, dipping around 20 weeks of gestation to 65-70 mm Hg, and rising through the end of gestation until median DBP levels are between 70-75 mm Hg.

Figure S3: Directed Acyclic Graph (DAG) Showing Hypothesized Relationships between Elevated Blood Pressure, Circulating EVP miRNA profiles during Pregnancy, and Covariates

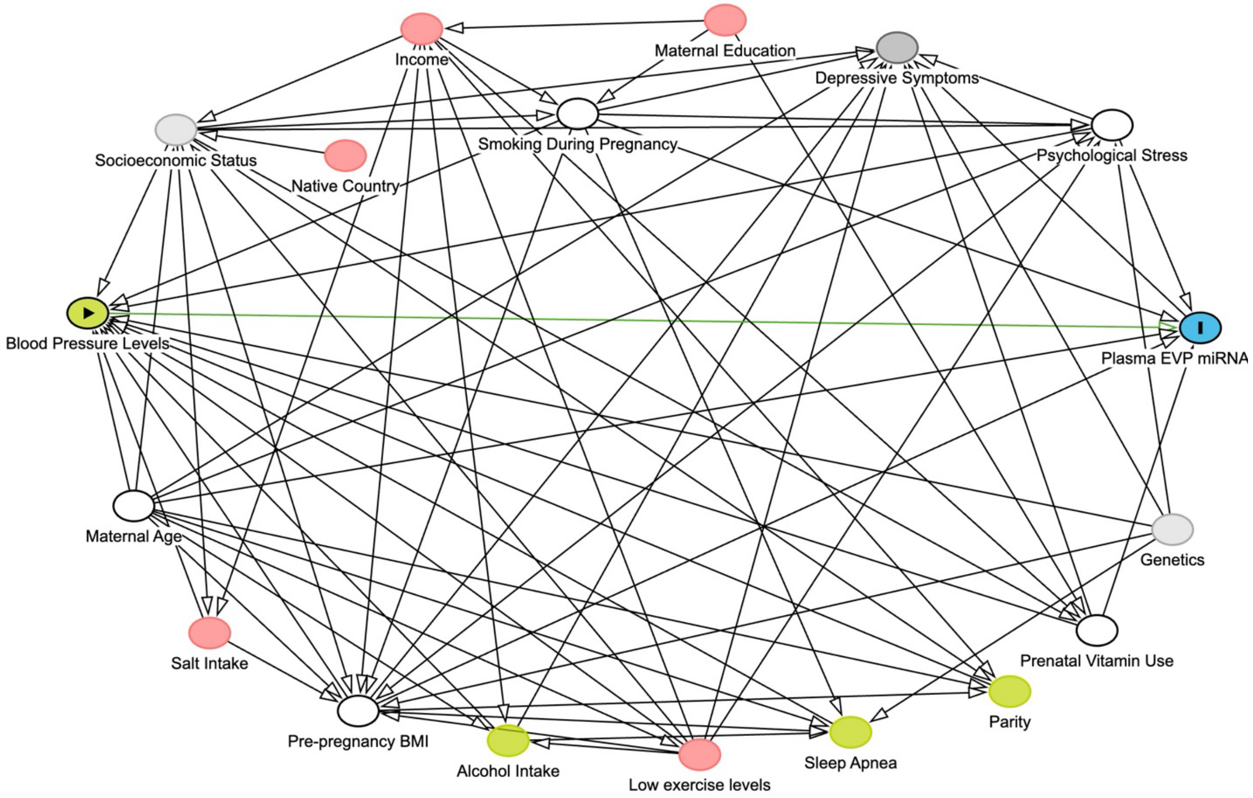

The DAG shown below was used to identify potential confounders of the association between elevated blood pressure levels and maternal circulating EVP miRNA levels during pregnancy. The minimal adjustment set identified by this DAG included maternal age, pre-pregnancy BMI, prenatal vitamin use, smoking during pregnancy, and psychological stress. There were no backdoor paths in the DAG after adjusting for the confounders in the minimal adjustment set.

Figure S4: Scree Plot of Plasma EVP miRNA PCs

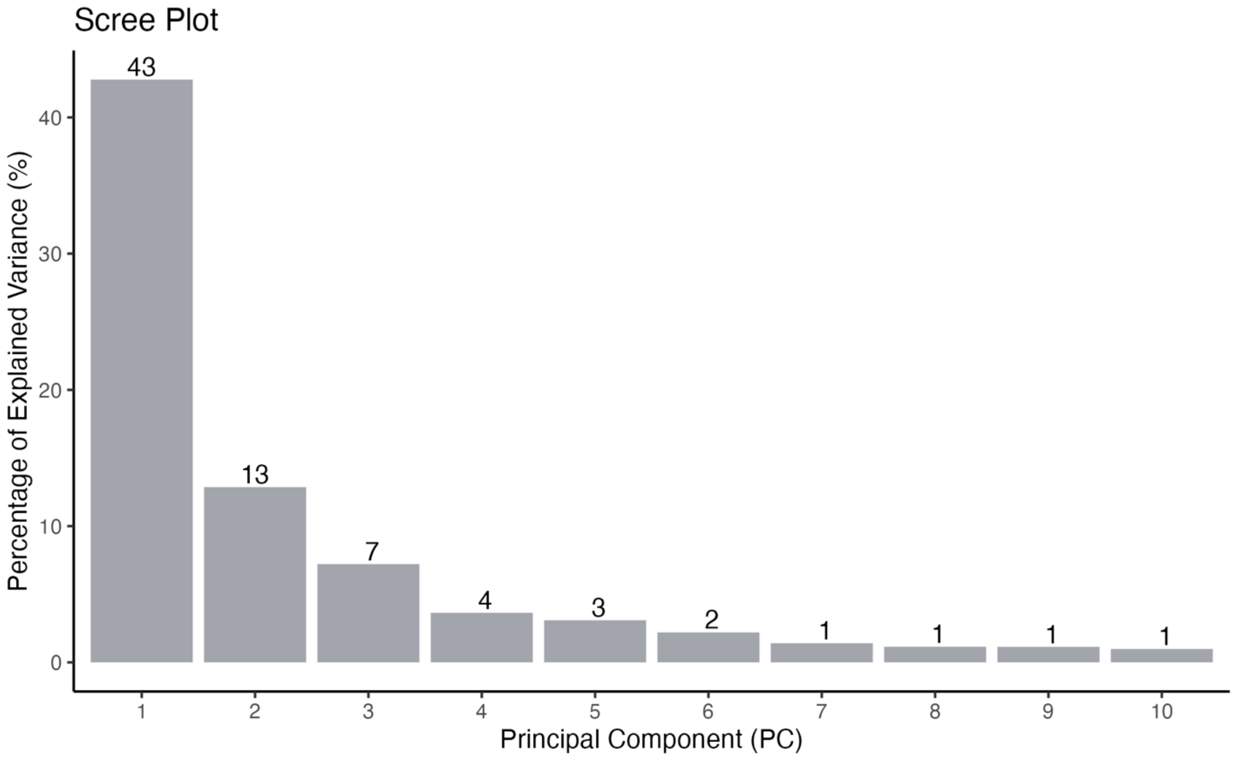

This scree plot shows the percentage of explained variance (y axis) for the top 10 Principal Components (PCs) from a PCA analysis of the plasma EVP miRNAs quantified using the NanoString nCounter platform. The top PC accounts for 43% of variance.

Figure S5: Systolic Blood Pressure Trajectories for the k=4 Model

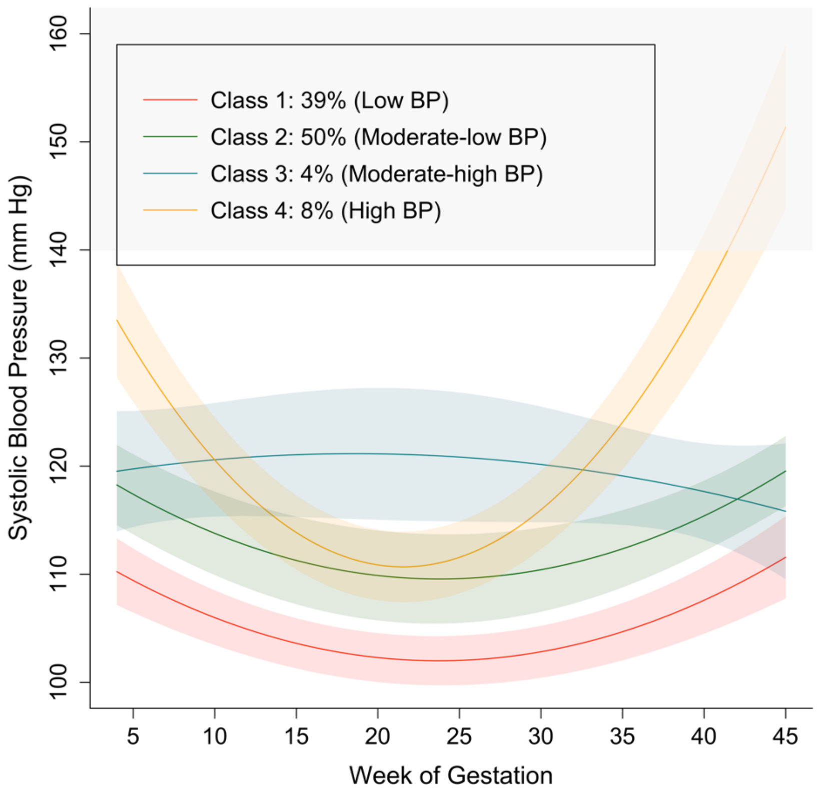

When latent class trajectory modeling was conducted without a random slope, we identified four systolic blood pressure (SBP) trajectories rather than three. These four trajectories are plotted with their median and 95% confidence interval bands: (1) Low BP, (2) Moderate-Low BP, (3) Moderate-High BP, and (4) High BP. The Moderate-Low BP class is unique to the k=4 model and features BP measures that on average are approximately 120 mm Hg in the beginning of pregnancy, then dip until reaching approximately 110 mm Hg at 20 weeks of gestation before rising until delivery.

Figure S6: Sankey Diagram of Participant Classification in the K=3 Trajectory Model vs. the K=4 Trajectory Model

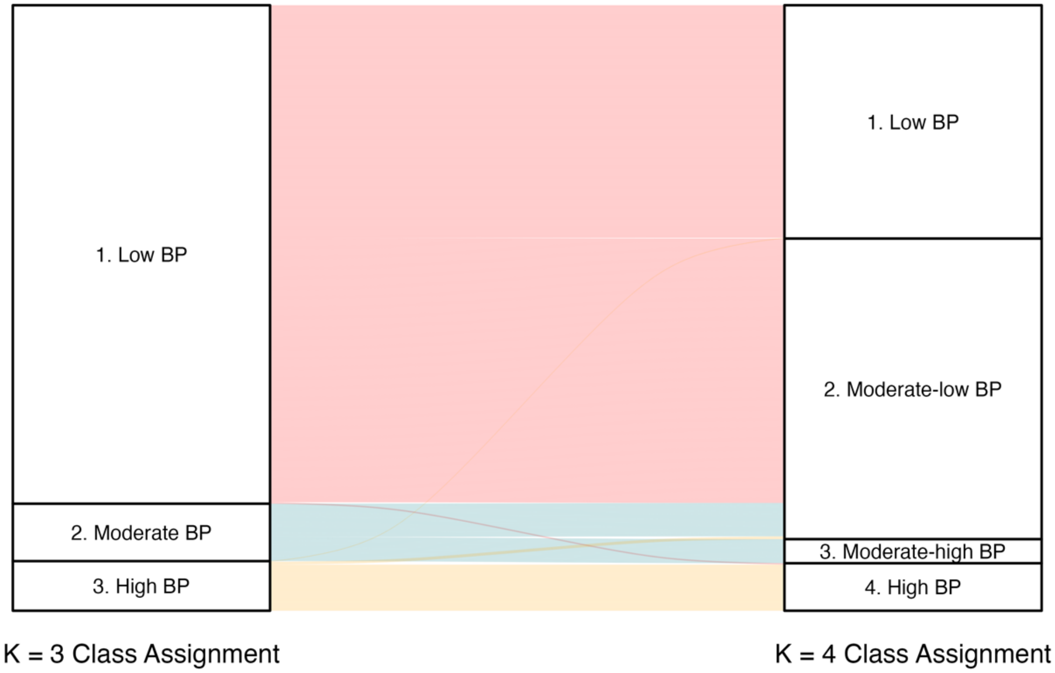

This Sankey diagram compares participant classification between the k=3 trajectory model (left) and the k=4 trajectory model (right). The class names on the left correspond to the class names in Figure 1, while the class names on the right correspond to the class names in Figure S2. The colors correspond with the trajectory colors shown in Figure 1. Participants classified as Low BP in the k=3 model are almost evenly split between the k=4 Low BP and Moderate-Low BP groups. Participants in the k=3 Moderate BP class are split between the k=4 Moderate-Low and Moderate-High BP groups. The majority (93%) of participants in the k=4 High BP trajectory were also classified as High BP in the k=3 model.

Figure S7: Venn Diagrams showing overlap of maternal circulating EVP miRNAs from the primary k=3 analyses and the k=4 sensitivity analyses

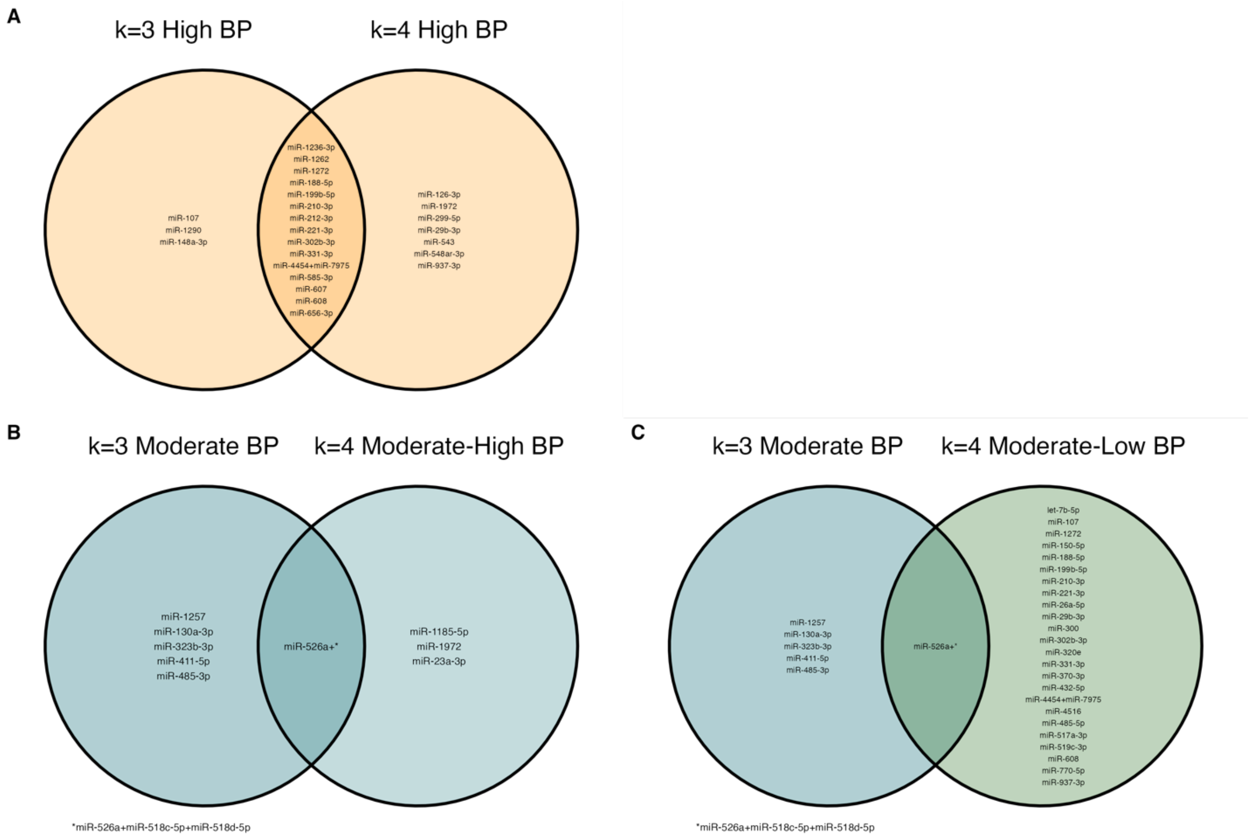

These diagrams compare the maternal circulating EVP miRNAs that were associated with each BP trajectory at $P < 0.05$ for the k=3 versus k=4 model. (A) Comparison of the miRNAs associated with the High BP trajectory identified using the k=3 vs. k=4 model. (B) Comparison of the miRNAs associated with the k=3 Moderate BP trajectory vs the k=4 Moderate-High BP trajectory. (C) Comparison of the miRNAs associated with the k=3 Moderate BP trajectory and the k=4 Moderate-Low BP trajectory.
